# When mtDNA COI is misleading: congruent signal of ITS2 molecular marker and morphology for North European *Melanostoma* Schiner, 1860 (Diptera, Syrphidae)

**DOI:** 10.3897/zookeys.431.7207

**Published:** 2014-08-06

**Authors:** Antti Haarto, Gunilla Ståhls

**Affiliations:** 1 Zoological Museum, Section of Biodiversity and Environmental Science, University of Turku, FI-20014 Turku, Finland; 2 Finnish Museum of Natural History, Zoological Museum, PO Box 17, FI-00014 University of Helsinki, Finland

**Keywords:** *Melanostoma*, taxonomy, ITS2, COI

## Abstract

The northern European taxa of genus *Melanostoma* Schiner, 1860 (Syrphidae, Diptera) are revised. A longstanding question concerning the number of *Melanostoma* taxa occurring in northern Europe prompted us to contrast and compare their morphological and molecular variability. Particular uncertainty concerned the putative existence of a sibling species of *Melanostoma mellinum*, and the identity of the taxon *Melanostoma dubium* in northern Europe due to existence of morphologically similar dark forms of *M. mellinum* in the northern parts of its distributional range. Partial sequences of two DNA markers, the mitochondrial protein-coding gene cytochrome *c* oxidase subunit I (COI-3') and the nuclear second internal transcribed spacer (ITS2) were analysed separately under parsimony. The obtained COI-3'gene fragment showed taxon-specific haplotypes and haplotypes that were shared among the taxa. The ITS2 sequences presented genotypes unique to each species, and congruence with our independently established taxonomic entities. Based on congruent signal of the ITS2 sequences and study of morphological characters we establish the presence of four taxa in northern Europe: *Melanostoma mellium* (= *M. dubium* nec auctt., **syn. n.**), *M. certum* **sp. n.** (= *M. dubium* auctt.), *M. mellarium* **stat. n.** (= *M. mellinum* auctt. partim) and *M. scalare*. Lectotype designations were made for *Musca mellina*, *Syrphus mellarius* and *Melanostoma mellinum* var. *melanatus*.

The following synonymies were established: *Melanostoma mellarium* = *Melanostoma melanatum* **syn. n.**; *Melanostoma mellinum* = *Scaeva dubia* **syn. n.**, *Melanostoma tschernovi* **syn. n.**, and *Melanostoma clausseni* **syn. n.** Morphological circumscriptions of the taxa and an identification key are presented.

## Introduction

The taxa of genus *Melanostoma* Schiner, 1860 (Diptera, Syrphidae, Syrphinae) are among the most abundant hoverflies in the northern Palaearctic region occurring in both undisturbed and human impacted woodlands and grasslands. The three presently recognized species on the European continent, *Melanostoma dubium* (Zetterstedt, 1838), *Melanostoma mellinum* (Linnaeus, 1758) and *Melanostoma scalare* (Fabricius, 1794) (Speight 1978), have long been understood and identified according to the key of van der Goot (1981). These three species are widely distributed in Europe, while two additional Palaearctic species belonging to the genus *Melanostoma*, *Melanostoma incompletum* Becker, 1908 and *Melanostoma wollastoni* Wakeham-Dawson, Franquinho-Aguiar, Smit, McCullough and Wyatt, 2004, are found endemic to the Canary islands (Spain) and island of Madeira (Portugal), respectively. Fauna Europaea lists an additional taxon, *Melanostoma pumicatum* (Meigen, 1838) (Speight 2004). We have seen digital images of the holotype female (deposited in MNHN) and this is a species of the genus *Platycheirus* Lepeletier & Serville, 1838. Barkalov (2009) described the taxa *Melanostoma clausseni* Barkalov, 2009 and *Melanostoma tschernovi* Barkalov, 2009 from Siberia, and stated that the species are morphologically close to *Melanostoma dubium*.

**Figure 1.**
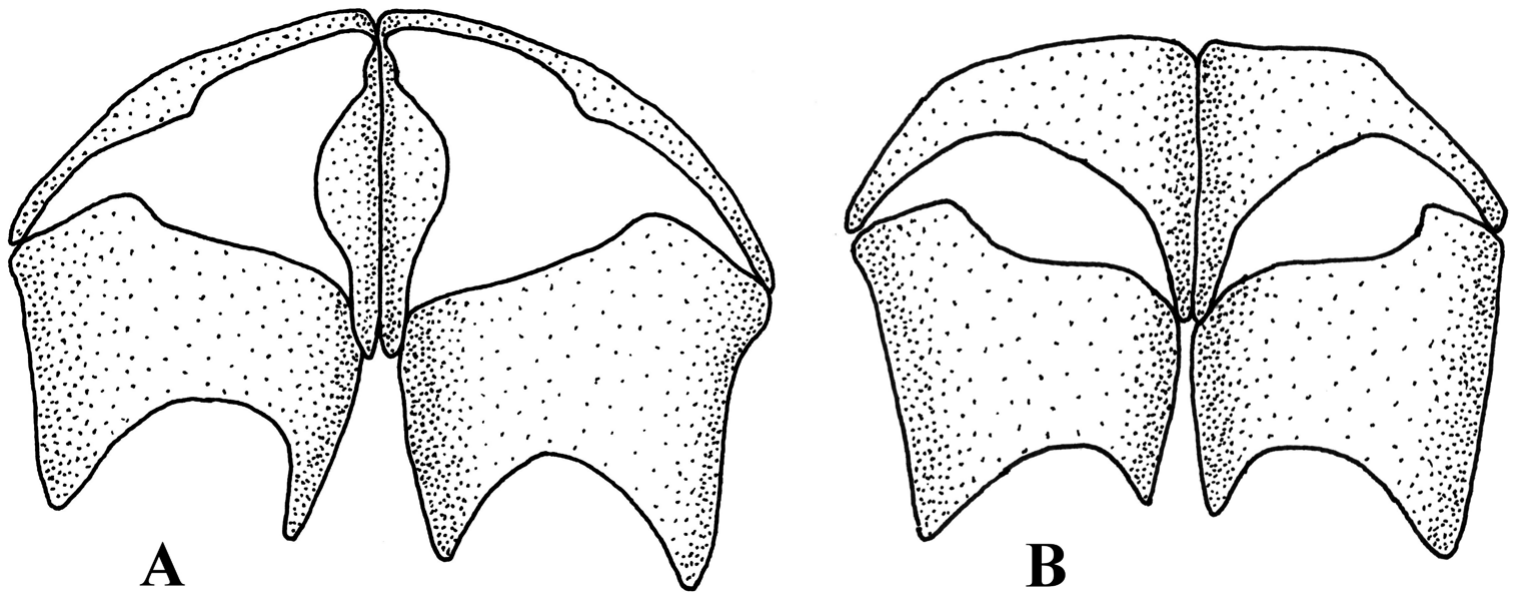
Shape of metasternum. **A** *Melanostoma mellarium* and **B** *Platycheirus podagratus*.

Species of the genus *Melanostoma* are medium-sized (5–9 mm) hover flies, dark coloured with a greenish or bluish tinge, usually with 1–4 pairs of variously shaped maculae on the abdomen. The genus *Melanostoma* is closely allied to genus *Platycheirus* Lepeletier & Serville, 1828. Both genera have bare eyes, a black face and scutellum, and antennae shorter than head. These genera have distinct shapes of surstyli and postgonites of the male genitalia (see e.g. Andersson 1970). Andersson (1970) was the first to identify the highly reduced metasternum (postero-lateral reduction so that the sclerotized portion consists of a median diamond-shaped area that readily differentiates taxa of genus *Melanostoma* from those of *Platycheirus* (Fig. 1). Additionally, in contrast to *Melanostoma*, most males of genus *Platycheirus* have modified protarsus and/or an apico-lateral curled pilis on profemur.

It has, however, long remained doubtful whether the present species definitions actually reflect the number of *Melanostoma* species in the continent (e.g. Speight 2006–2010). Recent central European faunistic works adhere to the established circumscriptions (e.g. Reemer et al. 2009), as do the recent faunistic works including identification keys by Haarto and Kerppola (2007) and Bartsch et al. (2009) treating the Fennoscandian hover fly fauna. All include the three *Melanostoma* taxa, but echo and stress an apparent need for a taxonomic study of the taxon. The colour variability and particularly lack of typical pale abdominal colour patterns of Nordic *Melanostoma mellinum* specimens result in frequent uncertainty at species identifications using the existing keys for such specimens. Kanervo (1934) named some North European morphologically aberrant forms, *Melanostoma mellinum* var. *angustatoides* (with large pale maculae on abdomen), *Melanostoma mellinum* var. *melanatus* (dark ‘melanic'abdomen) and *Melanostoma mellinum* var. *obscuripes* (unusually dark legs).

The present study attempts to finally resolve the longstanding confusion regarding the species identities, nomenclature and circumscriptions for the *Melanostoma* taxa occurring in northern Europe in light of not previously utilised molecular characters and informative new morphological characteristics. We employed DNA sequence characters of a large fragment of the 3'–end of the first subunit of the mitochondrial protein coding cytochrome c oxidase gene (hereafter COI) and of the nuclear second internal transcribed spacer region (ITS2). This allowed us to explore the congruence of the morphologically delimited species with the DNA haplotypes of COI and genotypes of ITS2 and to evaluate if our morphological hypotheses were supported by the molecular data. At the same time this approach allowed to contrast the usefulness (signal) of the employed molecular markers (COI and ITS2) for resolving the taxonomy of closely related hover fly species. The COI gene has been a work-horse both for taxonomic and systematic studies of invertebrates including Diptera, as the 5'–fragment of the gene constitutes the core barcoding gene region for animals (Hebert et al. 2003). The ITS2 region of the ribosomal rDNA cluster has been less explored at species level studies for insects. A study of Yao et al. (2010) indicated a species identification success rate of 91.7% for animals for the ITS2 marker, and that it unveiled a different ability to identify closely related species within and among different families and genera of both animals and plants. Marinho et al. (2011) stated that this marker was suitable both at species and generic level for Calliphoridae flies. This fast evolving spacer region was successfully used for tracking species boundaries of e.g. *Trichogramma* Westwood, 1833 parasitoids (Hymenoptera, Trichogrammatidae) (Ciociola et al. 2001), big-headed flies of subfamily Chalarinae (Diptera, Pipunculidae) (Kehlmaier and Assmann 2010), and hover fly taxa of the genus *Chrysotoxum* Meigen, 1803 (Masetti et al. 2006, Nedeljković et al. 2013).

## Material and methods

### Terminology

The characters used in the key, descriptions, and drawings employ the terminology established by Thompson (1999) and Cumming and Wood (2009). Index DL is the ratio of the distance between tip of projection and anterior edge of hypandrium to the length of projection (as shown for *Melanostoma scalare* in Fig. 10A).

### Type studies

The original label information of the examined type material is captured between single quotes ('’), and labels are separated with a slash /. Depository institutions of each specimen are indicated between square brackets after the label information. The acronyms used for collections largely follow the Evenhuis (2009) standards and their equivalents are as follow:

AHPC Antti Haarto personal collection, Turku, Finland

LSUK Linnean Collection of Insects, repository managed by the Linnean Society of London, London, UK

MNHN Muséum National d'Histoire Naturelle, Paris, France

MZH Finnish Museum of Natural History, Helsinki, Finland

MZL Museum of Zoology, Lund, Sweden

MZT Zoological Museum of the University of Turku, Finland

SKPC Sakari Kerppola personal collection, Helsinki, Finland

When necessary, a lectotype has been designated and labelled accordingly in order to fix the concept of the taxon in question and to ensure the universal and consistent interpretation of the same.

### Images

Images of external morphology (pinned specimens; 30–40 exposures; Canon EOS 40S digital camera) and male genitalia (submersed in ethanol; 20–30 exposures; Olympus E520 digital camera on Olympus S7X16 microscope) were taken using d-cell software vs 5.1 and composed using CombineZP software vs. 2 (Hadley 2010).

### Taxon sampling for morphological study

In addition to the DNA voucher specimens, abundant material of pinned flies of the *M. mellinum sensu lato* (112 males, 138 females) and *Melanostoma scalare* taxa (27 males, 25 females), from localities in northern Europe (coll. MZH, MZT and AHPC), were available for study of morphological characteristics (Table 1). For *Melanostoma dubium* only about ten pinned specimens of each sex were obtained for this study (coll. MZH, MZT and AHPC), including the DNA vouchers.

### Taxon sampling for molecular study

A comprehensive sample of specimens identified according to present concepts as *Melanostoma dubium*, *Melanostoma mellinum* and *Melanostoma scalare* from Fennoscandia were used for molecular work. Additionally, specimens of *Melanostoma mellinum* auctt. and *Melanostoma scalare* obtained from a broad geographical range across Europe were also available for molecular work, including one sample of *Melanostoma incompletum* from the Canary Islands (Table 1). Specimens of *M. dubium, M. tschernovi*, *Melanostoma clausseni* and *Melanostoma mellinum* from northern Siberia, Russia (provided and identified by A. V. Barkalov) were also subjected to molecular analyses. Specimens used for molecular study are listed in Table 1. Locality labels for samples from Finland include Finnish grid coordinates (ykj) (see http://www.maanmittauslaitos.fi/sites/default/files/Finnish_Coordinate_Systems.pdf). Added geographical coordinates in DMS are shown in square brackets. DNA voucher specimens were deposited in the MZH and labelled accordingly.

### Laboratory procedures

DNA was extracted from 1–3 legs of dry pinned or ethanol preserved specimens using the Nucleospin Tissue DNA extraction kit (Machery-Nagel, Düren, Germany) following the manufacturer’s protocols and then re-suspended in 50 µl of ultra-pure water. PCR reactions were carried out using GE Ready-to-Go PCR beads in 25 µl reaction aliquots containing 2–4 µl DNA extract, 1 µl of each primer (at 10 pmol/µl) and ultrapure water. Thermocycler conditions were initial denaturing at 95°C 2 min, 29 cycles of 30 s denaturing at 94°C, 30 s annealing at 49°C, 2 min extension at 72°C, followed by a final extension of 8 min at 72°C. The universally conserved primers used for amplifying and sequencing the COI 3'–fragment (ca 770 bp) were the forward primer C1-J-2183 [5'–CAACATTTATTTTGATTTTTTGG–3'] (alias JERRY) and reverse primer TL2-N-3014 [5'–TCCAATGCACTAATCTGCCATATTA–3'] (alias PAT) (Simon et al. 1994), and the primers ITS2a [5'–TGTGAACTGCAGGACACAT–3'] and ITS2b [5'–TATGCTTAAATTCAGGGGGT–3'] (Beebe and Saul 1995) for the ITS2 marker. The ITS2 marker was only amplified for specimens of less than 3 years old, as older samples failed.

Amplified PCR products were electrophoresed on 1.5% agarose gels and treated with Exo-SapIT (USB Affymetrix, Ohio, USA) prior to sequencing. Both PCR primers were used for sequencing. The Big Dye Terminator Cycle Sequencing Kit (version 3.1) (Applied Biosystems, Foster City, CA, USA) was used on an ABI 3730 (Applied Biosystems, Foster City, CA, USA) genetic analyzer at the Sequencing Service Laboratory of the Finnish Institute for Molecular Medicine (ww.fimm.fi). The sequences were edited for base-calling errors and assembled using Sequencher™ (version 4.9) (Gene Codes Corporation, Ann Arbor, MI, USA). All new sequences were submitted to GenBank (see Table 1 for accession numbers).

### Sequence alignment

The protein-coding COI gene was aligned manually and it was not necessary to include gaps in this alignment. The alignment of the ITS2 fragment was carried out using the E-INS-I strategy as implemented in MAFFT (Katoh et al. 2005, 2009).

### DNA sequence analyses

*Platycheirus europaeus* (Goeldlin, Maibach & Speight, 1990) (Diptera, Syrphidae) was used to root the trees. Single gene parsimony analyses were conducted for each gene region. Parsimony analysis was performed using NONA (Goloboff 1999) and spawn with the aid of Winclada (Nixon 2002), using heuristic search algorithm with 1000 random addition replicates (mult*1000), holding 10 trees per round (hold/10), max trees set to 10000 and applying TBR branch swapping. All base positions were treated as equally weighted characters, and gaps were treated as unknown. Nodal support was assessed with bootstrap resampling (1000 replicates) using Winclada (Nixon 2002).

**Table 1.** List of specimens used for molecular work including GenBank accession numbers.

Labcode	Taxon	Country	Finnish grid coordinates and /or geogr. coordinates	Province	Locality	Date	Collector	GenBank accession mtDNA COI	GenBank accession ITS2
MZH_Y397	*Melanostoma certum*	Finland	7674:3253 69°2.59'N, 20°48.316'E	Le: Enontekiö	Kilpisjärvi	6.VII.2005	G. Ståhls & V. Milankov leg.	KJ848068	KJ847974
MZH_Y472	*Melanostoma certum*	Finland	7588:3334 68°20'N, 22°58"E	Le: Enontekiö	Vähäniva	16.VI.2006	E.M. & L. Laasonen leg.	KJ848069	KJ847975
MZH_Y491	*Melanostoma certum*	Finland	77224:34611 69°34'42"N, 25°59'49"E	Li: Inari	Akujoen risteys	2.VII.2006	I. Kakko leg.	NA	KJ847976
MZH_Y643	*Melanostoma certum*	Finland	76865:2790 69°10'9"N, 21°25'22"E	Le: Enontekiö	Annjaloanji	13.VII.2007	A. Haarto leg.	NA	KJ847977
MZH_Y1648	*Melanostoma certum*	Finland	76865:2790 69°10'9"N, 21°25'22"E	Le: Enontekiö	Annjaloanji	22.VII.2005	A. Haarto leg.	NA	KJ847978
MZH_Y1872	*Melanostoma certum*	Finland	77042:34600 69°24'54"N, 25°58'36"E	Li: Utsjoki	Karigasniemi, Ailigas	25.VI.2013	K. Mattila leg.	KJ848107	KJ847979
MZH_Y395	*Melanostoma mellarium*	Finland	7674:3253	Le: Enontekiö	Kilpisjärvi	6.VII.2005	G. Ståhls & V. Milankov leg.	KJ848078	KJ847981
MZH_Y396	*Melanostoma mellarium*	Finland	7674:3253	Le: Enontekiö	Kilpisjärvi	6.VII.2005	G. Ståhls & V. Milankov leg.	NA	KJ847982
MZH_Y407	*Melanostoma mellarium*	Finland	729:38:00	Lkem: Kemi	Ajos	15.VI.2004	E.M. & L. Laasonen leg.	NA	KJ847983
MZH_Y415	*Melanostoma mellarium*	Finland	67071:090	Al: Eckerö	Skag	1.VI.2005	E.M. & L. Laasonen leg.	KJ848079	KJ847985
MZH_Y416	*Melanostoma mellarium*	Finland	708:38	Ob: Sievi	Kiiskilä	17.VI.2005	E.M. & L. Laasonen leg.	KJ848080	KJ847986
MZH_Y417	*Melanostoma mellarium*	Finland	708:38	Ob: Sievi	Kiiskilä	17.VI.2005	E.M. & L. Laasonen leg.	NA	KJ847987
MZH_Y438	*Melanostoma mellarium*	Finland	67549:35144	Sa: Luumäki	Päivärinne	9.VI.2006	J. Kahanpää leg.	NA	KJ847988
MZH_Y439	*Melanostoma mellarium*	Finland	67549:35144	Sa: Luumäki	Päivärinne	9.VI.2006	J. Kahanpää leg.	KJ848081	KJ847989
MZH_Y453	*Melanostoma mellarium*	Finland		Li: Inari	KJ	8.VII.2005	E.M. & L. Laasonen leg.	NA	KJ847992
MZH_Y527	*Melanostoma mellarium*	Norway	EIS 160	FO Sör-Varanger	Pasvik, Skogfoss	28.VI.2006	T. R. Nielsen leg.	KJ848083	KJ847993
MZH_Y530	*Melanostoma mellarium*	Norway	EIS 160	FO Sör-Varanger	Pasvik, Skogfoss	29.VI.2006	T. R. Nielsen leg.	KJ848084	KJ847994
MZH_Y531	*Melanostoma mellarium*	Norway	EIS 160	FO Sör-Varanger	Pasvik, Fagermo	28.VI.2006	T. R. Nielsen leg.	NA	KJ847995
MZH_Y612	*Melanostoma mellarium*	Finland	76178:35210	Li: Ivalo	Näveriniemi	5.VII.2007	G. Ståhls leg.	NA	KJ847990
MZH_Y621	*Melanostoma mellarium*	Finland	77586:35009	Li: Utsjoki	roadside	8.VII.2007	G. Ståhls leg.	KJ848095	KJ847996
MZH_Y642	*Melanostoma mellarium*	Luxembourg		Bonnerue meadow	L’Ourtie occidental, 229-78	21.V.2006	W. van Steenis leg.	KJ848091	KJ847991
MZH_Y646	*Melanostoma mellarium*	Finland	76764:2523	Le: Enontekiö	Saana (koivikko)	16.VII.2007	A. Haarto leg.	NA	KJ847997
MZH_Y647	*Melanostoma mellarium*	Finland	76764:2523	Le: Enontekiö	Saana (koivikko)	16.VII.2007	A. Haarto leg.	KJ848092	KJ847998
MZH_Y770	*Melanostoma mellarium*	Norway	EIS 169	FÖ Sör-Varanger	Svanvik	30.VI.2008	T. R. Nielsen leg.	NS	KJ847984
MZH_Y1650	*Melanostoma mellarium*	Finland	775:350	Le: Enontekiö	Saana	11.VII.2011	E.M. & L. Laasonen leg.	NA	KJ847980
MZH_Y399	*Melanostoma mellinum*	Finland	6771:255	Ta: Lammi	Biol. station	28.V.2005	G. Ståhls leg.	NA	KJ847999
MZH_Y400	*Melanostoma mellinum*	Finland	6771:255	Ta: Lammi	Biol. station	28.V.2005	G. Ståhls leg.	NA	KJ848000
MZH_Y405	*Melanostoma mellinum*	Finland	6682:108	Al: Mariehamn	Espholm	30.V.2005	E. M. & L. Laasonen leg.	NA	KJ848001
MZH_Y406	*Melanostoma mellinum*	Finland	7623:539	Li: Inari	Heinäj.	7.VII.2005	E.M. & L. Laasonen leg.	NA	KJ848002
MZH_Y409	*Melanostoma mellinum*	Finland		Li: Utsjoki	Tsuomas	5–6.VII.2005	E.M. & L. Laasonen leg.	NA	KJ848003
MZH_Y410	*Melanostoma mellinum*	Finland	69103:5353	Ta: Joroinen		29.VI.2006	A. Haarto leg.	KJ848072	KJ848004
MZH_Y413	*Melanostoma mellinum*	Finland	6696:124	Al: Sund	Bomarsund	1.VI.2005	E.M. & L. Laasonen leg.	NA	KJ848005
MZH_Y414	*Melanostoma mellinum*	Finland	6696:124	Al: Sund	Bomarsund	1.VI.2005	E.M. & L. Laasonen leg.	NA	KJ848006
MZH_Y419	*Melanostoma mellinum*	Finland		Li: Kiilopää		16–24.VII.2005	E.M. & L. Laasonen leg.	NA	KJ848007
MZH_Y434	*Melanostoma mellinum*	Finland	6696:234	Ab: Parainen	Petteby	31.V.2006	A. Haarto leg.	NA	KJ848008
MZH_Y435	*Melanostoma mellinum*	Finland	6733:222	Ab: Mietoinen	Perkko	28.V.2006	A. Haarto leg.	NA	KJ848009
MZH_Y436	*Melanostoma mellinum*	Finland	6696:234	Ab: Parainen	Petteby	31.V.2006	A. Haarto leg.	KJ848087	KJ848010
MZH_Y437	*Melanostoma mellinum*	Finland	6733:222	Ab: Mietoinen	Perkko	25.V.2006	A. Haarto leg.	NA	KJ848011
MZH_Y442	*Melanostoma mellinum*	Finland		Ok: Kuhmo	Härkäniementie	18.VIII.2006	G. Ståhls leg.	KJ848071	KJ848012
MZH_Y451	*Melanostoma mellinum*	Finland		Le: Kilpisjärvi		VII.2005	G. Ståhls leg.	NA	KJ848013
MZH_Y452	*Melanostoma mellinum*	Finland	7747:472	Li: Utsjoki		30.VI.2005	E.M. & L. Laasonen leg.	NA	KJ848014
MZH_Y456	*Melanostoma mellinum*	Sweden		Uppland	Järfälla	VIII.2006	H. Bartsch leg.	KJ848085	KJ848015
MZH_Y457	*Melanostoma mellinum*	Sweden		Uppland	Järfälla	VIII.2006	H. Bartsch leg.	KJ848074	KJ848016
MZH_Y475	*Melanostoma mellinum*	Finland	664:18	Ab: Korpo	Utö	28.7.2006	A. Haarto leg.	NA	KJ848017
MZH_Y479	*Melanostoma mellinum*	Netherlands	RD 128-566	Breukelen	Overholland	5.V.2006	W. van Steenis leg.	KJ848086	KJ848018
MZH_Y480	*Melanostoma mellinum*	Netherlands	RD 128-464	Breukelen	Niejenrode	5.V.2006	W. van Steenis leg.	KJ848075	KJ848019
MZH_Y488	*Melanostoma mellinum*	Finland	77042:34600	Li: Utsjoki	Kaivojoki, Karigasniemi	01.VII.2006	I. Kakko leg.	KJ848076	KJ848020
MZH_Y489	*Melanostoma mellinum*	Finland	76954:34829	Li: Utsjoki	Kaamasmukka	01.VII.2006	I. Kakko leg.	KJ848077	KJ848021
MZH_Y490	*Melanostoma mellinum*	Finland	77224:34611	Li: Inari	Akujoen risteys	02.VII.2006	I. Kakko leg.	NA	KJ848022
MZH_Y528	*Melanostoma mellinum*	Norway	EIS 160	FO Sör-Varanger	Pasvik, Skogfoss	27.VI.2006	T. R. Nielsen leg.	KJ848073	KJ848023
MZH_Y529	*Melanostoma mellinum*	Norway	EIS 160	FO Sör-Varanger	Pasvik, Skogfoss	28.VI.2006	T. R. Nielsen leg.	NA	KJ848024
MZH_Y593	*Melanostoma mellinum*	Italy		Sardinia	Prov. Sassari	3.VI.2007	C. Kehlmaier leg.	NA	KJ848025
MZH_Y611	*Melanostoma mellinum*	Finland	75944:35160	Li: Saariselkä	Kaunispäänoja	5.VII.2007	G. Ståhls leg.	NA	KJ848034
MZH_Y613	*Melanostoma mellinum*	Finland	77426:35005	Li: Utsjoki	Kevo, Kutuniemi	9.VII.2007	G. Ståhls leg.	NA	KJ848035
MZH_Y614	*Melanostoma mellinum*	Finland	77422:34997	Li: Utsjoki	Kevonsuu	11.VII.2007	G. Ståhls leg.	NA	KJ848036
MZH_Y619	*Melanostoma mellinum*	Finland	77586:35009	Li: Kaunispäänoja	roadside	5.VII.07	A. Ssymank leg.	NA	KJ848026
MZH_Y620	*Melanostoma mellinum*	Finland	76872:2807	Li: Utsjoki	roadside	8.VII.2007	G. Ståhls leg.	KJ848094	KJ848027
MZH_Y644	*Melanostoma mellinum*	Finland	76865:2790	Le: Enontekiö	Toskaljoki	16.VII.2007	A. Haarto leg.	NA	KJ848029
MZH_Y645	*Melanostoma mellinum*	Finland	76764:2523	Le: Enontekiö	Annjaloanji	13.VII.2007	A. Haarto leg.	NA	KJ848030
MZH_Y647	*Melanostoma mellarium*	Finland	7678:251	Le: Enontekiö	Saana (koivikko)	16.VII.2007	A. Haarto leg.	NA	NA
MZH_Y648	*Melanostoma mellinum*	Finland	7678:251	Le: Enontekiö	Siilasjärvi	11.VII..2007	A. Haarto leg.	KJ848093	KJ848031
MZH_Y649	*Melanostoma mellinum*	Finland	7678:251	Le: Enontekiö	Siilasjärvi	11.VII..2007	A. Haarto leg.	NA	KJ848032
MZH_Y697	*Melanostoma mellinum*	Finland	7594:516	Li: Inari	K-oja	5.VII..2007	E.M. & L. Laasonen leg.	KJ848070	KJ848033
MZH_Y770	*Melanostoma mellinum*	Norway	EIS 169	FÖ Sör-Varanger	Svanvik	30.VI.2008	leg. T.R. Nielsen	KJ848099	KJ848037
MZH_Y1586	*Melanostoma mellinum*	Finland	75916:38516	Li: Inari		30.VI.2011	E.M. & L. Laasonen leg.	NA	KJ848038
MZH_Y1613	*Melanostoma mellinum*	Finland	75916:85162	Li: Inari		30.VI.2011	E.M. & L.Laasonen leg.	NA	KJ848039
MZH_Y1625	*Melanostoma mellinum*	Russia		Taimyr	114 km from Khatangi at river Kotyi	22.VI.2010	A.V. Barkalov leg.	KJ848096	KJ848040
MZH_Y1626	*Melanostoma mellinum*	Russia		Taimyr	114 km from Khatangi at river Kotyi	22.VI.2010	A.V. Barkalov leg.	KJ848097	KJ848041
MZH_Y1627	*Melanostoma dubium*	Russia		Taimyr	114 km from Khatangi at river Kotyi	22.VI.2010	A.V. Barkalov leg.	KJ848098	KJ848042
MZH_Y1628	*Melanostoma dubium*	Russia		Taimyr	114 km from Khatangi at river Kotyi	22.VI.2010	A.V. Barkalov leg.	NA	KJ848043
MZH_Y1629	*Melanostoma clausseni* Barkalov, 2009 Paratype	Russia		republ. Altai	Ulaganskij raion	1–4.VII.2008	R. Dudko leg.	NA	KJ848060
MZH_Y1630	*Melanostoma tschernovi* Barkalov, 2009	Russia		Taimyr	Shore of Zakharova Rassocha	3.VII.2011	A. V. Barkalov leg.	NA	KJ848061
MZH_Y1631	*Melanostoma tschernovi* Barkalov, 2009	Russia		Taimyr	Shore of Zakharova Rassocha	3.VII.2011	A. V. Barkalov leg.	NA	KJ848063
MZH_Y1660	*Melanostoma mellinum*	Greece		Olymp		18.V.2011	A. Vujic leg.	KJ848102	KJ848044
MZH_Y1661	*Melanostoma mellinum*	Greece		Samos		17.IV.2011	A. Vujic & S. Radenkovic leg.	KJ848103	KJ848045
MZH_Y1662	*Melanostoma mellinum*	Serbia		Tara		5.VIII.2010	A. Vujic leg.	KJ848105	KJ848046
MZH_Y1664	*Melanostoma mellinum*	Greece		Olymp		18.V.2011	A. Vujic leg.	KJ848104	KJ848047
MZH_Y1785	*Melanostoma tschernovi* Barkalov, 2009	Russia	73°24'N, 80°39'E	NW Tajmyr península		6.VII.2012	A.V. Barkalov leg.	NA	KJ848065
MZH_Y1786	*Melanostoma tschernovi* Barkalov, 2009	Russia	73°24'N, 80°39'E	NW Tajmyr península		16.VII.2012	A.V. Barkalov leg.	KJ848100	KJ848064
MZH_Y1871	*Melanostoma mellinum*	Turkey		Bozdag mnt		16.IX.2013	G. Ståhls leg.	KJ848106	KJ848056
MZH_Y1880	*Melanostoma mellinum*	Russia		Chukotka,	near river Anadyr	23.VII.2013	A. V. Barkalov leg.	KJ848101	KJ848059
MZH_E61	*Melanostoma mellinum*	Cyprus		Almirolibado		31.V.–2.VI.2012	S. Dimitriou leg.	KJ848109	KJ848057
MZH_E62	*Melanostoma mellinum*	Cyprus		Almirolibado		31.V.–2.VI.2012	S. Dimitriou leg.	KJ848108	KJ848058
MZH_Y398	*Melanostoma scalare*	Finland	7663:149	Le: Kilpisjärvi		6.VII.2005	G. Ståhls & V. Milankov leg.	NA	KJ848048
MZH_Y401	*Melanostoma scalare*	Hungary		W Somlo	Weingut	10.IX.2005	E.M. & L. Laasonen leg.	NA	KJ848052
MZH_Y402	*Melanostoma scalare*	Finland	68744:5732	Ta: Rantasalmi,	Hiltula	30.VI.2006	A. Haarto leg.	NA	KJ848049
MZH_Y403	*Melanostoma scalare*	Finland	68744:5732	Ta: Rantasalmi,	Hiltula	30.VI.2006	A. Haarto leg.	NA	KJ848050
MZH_Y404	*Melanostoma scalare*	Finland	6682:108	Al: Mariehamn	Espholm	30.V.2005	E. Laasonen leg.	NA	KJ848051
MZH_Y441	*Melanostoma scalare*	Finland		Ok: Kuhmo	Lentuankoski	15.VIII.2006	G. Ståhls leg.	KJ848082	KJ848053
MZH_Y594	*Melanostoma scalare*	Italy	8°35'586"E, 40°10'377"N	Sardinia	Prov. Oristano, Il Montiferru	8.IV.2007	leg. C. Kehlmaier	KJ848090	KJ848055
MZH_Y641	*Melanostoma scalare*	Netherlands	RD 128-463	Breukelen	Nijenrode	23.IV.2006	W. van Steenis leg.	KJ848089	KJ848054
MZH_Y1838	*Melanostoma incompletum* Becker, 1908	Spain		Canary islands	Tenerife, 3 km S Los Realejos	16.II.2013	M. Reemer leg.	NA	KJ848066
MZH_Y443	*Platycheirus europaeus* Goeldlin, Maibach & Speight, 1990	Finland		Ok: Kuhmo	Lentuankoski	15.VIII.2006	G. Ståhls leg.	KJ848067	KJ847973

## Results

### Type studies

Due to the geographic and taxonomic focus of the present study, the following type material of *Melanostoma dubium* and *Melanostoma mellinum* and part of their currently recognised synonyms were studied.

***Melanostoma mellinum* (Linnaeus, 1758)**

Peck (1988) listed altogether 18 synonyms for *Melanostoma mellinum*. We do accept all of these synonyms and give some notes on the studied taxa.

*Musca mellina* Linnaeus, 1758

Thompson et al. (1982) in their review of the Linnaean species of flower flies (Diptera, Syrphidae) restricted the type locality of the taxon to Sweden. They indicated that four female specimens were present in the Linnaean collection, of which two specimens are different *Platycheirus* species, another one corresponds to *Melanostoma scalare* and the last one to *Melanostoma mellinum* auctt. They accepted all specimens as syntypes and did not choose a lectotype for the *Melanostoma mellinum* taxon. We designate the specimen with collection number LINN 5304 as lectotype of *Musca mellina* Linnaeus, 1758 and have labelled it accordingly [in LSUK].

*Syrphus mellarius* Meigen, 1822

This taxon was described based on an unknown number of males and females. The type locality is “Nord de la France”. In MNHN collections two female syntype specimens exist, one with labels ’Meigen 1486 40 / Syrphus mellarius female'and another female labelled ’Meigen 1486 40 / mellinum type’. The first mentioned female is a specimen of *M. scalare.* We herewith designate the second female as the lectotype of *Syrphus mellarius* Meigen, 1822 and have labelled it accordingly. Based on our results (see below) it is hereafter named *Melanostoma mellarium* (Meigen, 1822), stat. n.

*Syrphus melliturgus* Meigen, 1822

Type locality “Nord de la France”. In MNHN, only one specimen remains labelled ’Meigen 1482 40 / *Syrphus melliturgus* male’. Of the pinned specimen only the thorax with legs and both wings remains. The identity of the specimen cannot be ascertained.

*Syrphus minutus* Macquart, 1829

The taxon was described based on a single male. The type locality was not given. The type was not found at MNHN, but apparently exists in the collections of Musée d’Histoire Naturelle de Lille, France (curator P. de Bleeckere, pers. comm.) where some Macquart types remain/were deposited. Type material was not available for this study.

*Syrphus unicolor* Macquart, 1829

Macquart mentioned several females, with black abdomen. The type locality was not given. Syntypes were not found in MNHN, but an unknown number of syntypes apparently exist in the collections of Musée d’Histoire Naturelle de Lille, France (curator P. de Bleeckere, pers. comm.) where some Macquart types remain/were deposited. Syntypes could not actually be studied.

*Melanostoma mellinum* var. *angustatoides* Kanervo, 1934

Kanervo (1934) described three varieties (“Varietäten”) of *Melanostoma mellinum* (deposited in MZT) and listed additional specimens of two of the varieties in later publications (Kanervo 1938a, 1938b). *Melanostoma mellinum* var. *angustatoides* was described based on one male from Sodankylä (Finland), but this specimen could not be located in MZT and is presumably lost.

*Melanostoma mellinum* var. *melanatus* Kanervo, 1934

This taxon was recognized based on three females from Haukilampi locality (Murmansk region, Russia) with “completely melanic abdomen”. We have studied the two female specimens found in MZT and labelled one as lectotype. The lectotype has the following labels: ‘Haukilampi, 28.4.28'/ ‘Lectotype *Melanostoma mellinum* var. *melanatus* Kanervo, Haarto & Ståhls des. 2014’. Both specimens belong to *Melanostoma mellarium* (syn. n.).

*Melanostoma mellinum* var. *obscuripes* Kanervo, 1934

Kanervo (1934) indicated a single female holotype of *Melanostoma mellinum* var. *obscuripes* from Parkkino (near Pechenga, Murmansk region, Russia), but in MZT only a male was found with this data, which also represents *Melanostoma mellarium*. The holotype is presumably lost. The taxon cannot be placed in synonymy with certainty.

***Melanostoma dubium* (Zetterstedt, 1838)**

In the Catalogue of Palaearctic Diptera, Peck (1988) listed four synonyms of *Melanostoma dubium*.

*Scaeva dubia* Zetterstedt, 1838

The nominate form, var. a, was described based on females from Torne lappmark, Lycksele lappmark and Åsele lappmark, northern Sweden. A second form, var. b, was described based on females from Lycksele lappmark, northern Sweden, and Finnmark, northern Norway. Andersson (1970) designated a female from the nominate series labelled ’S. dubia f Juckasjärvi'[= Torne lappmark] as lectotype. We have examined the lectotype and the three var. a syntypes deposited in MZL. The lectotype is labelled ‘S. dubia ♀, Juckasjärv.'[handwritten] / Lectotypus Scaeva dubia Zett. [printed red label].One of the syntypes is *Melanostoma mellinum* (original label ‘Lapp. Lyck.'[handwritten]), and the other two female syntypes belong to *Platycheirus hyperboreus* (Staeger, 1845) (one lacking written label, only with very small black colour label, the other syntype with original label ‘Åsele 27 Jul.’[handwritten]). Also, we examined the two female syntypes of the *Scaeva dubia* var. b present at MZL. These two females belong to *Platycheirus europeus* (lacking written label, only with very small black colour label) and *Platycheirus podagratus* (Zetterstedt, 1838) (original label ‘var. b. ♀ altern. [handwritten, last word unclear], respectively. Thus, *Scaeva dubia* (= *Melanostoma dubium* nec auctt.) is a junior synonym of *Melanostoma mellinum* syn. n.

*Syrphus unicolor* Rondani, 1857

This is a junior primary homonym of *Syrphus unicolor* Macquart, 1829.

*Chilosia (Anocheila) freyi* Hellén, 1949

This taxon was described from northern Finland. The holotype female deposited in MZH is damaged, only thorax, wings and legs remain. It certainly is a species of *Melanostoma*, but we are unable to identify this taxon with certainty.

*Pachyspyria flavitibia* Enderlein, 1938 and *Pachyspyria sexpunctatum* Enderlein, 1938 were described as variations of *Scaeva dubia*. Both species names accent morphological characteristics (yellow tibiae, and abdomen with six maculae/patches) that are not diagnostic for *Melanostoma dubium* auctt. Furthermore, their type localities are central European. The type materials were not studied by us. The names cannot be accepted as synonyms of *Melanostoma dubium* auctt., and we cannot place them in synonymy with any *Melanostoma* taxon.

Accordingly, the taxon identified by authors as *Melanostoma dubium* (*Melanostoma dubium* auctt. nec Zetterstedt) is a different taxon from the *Melanostoma mellinum* taxon. The lectotype of *Melanostoma dubium* (Zetterstedt) is a synonym of *Melanostoma mellinum*. Taking the above presented information into consideration, the *Melanostoma dubium* auctt. nec Zetterstedt taxon is in need of a new name and type designation.

### Molecular studies

We successfully obtained mtDNA COI sequences for 41 ingroup *Melanostoma* specimens with 743 bp unambiguous sequence alignment, and ITS2 for 93 ingroup terminals with sequence length variation among all ingroup taxa between 400–404 bp with a total dataset alignment of 409 bp.

**Figure 2.**
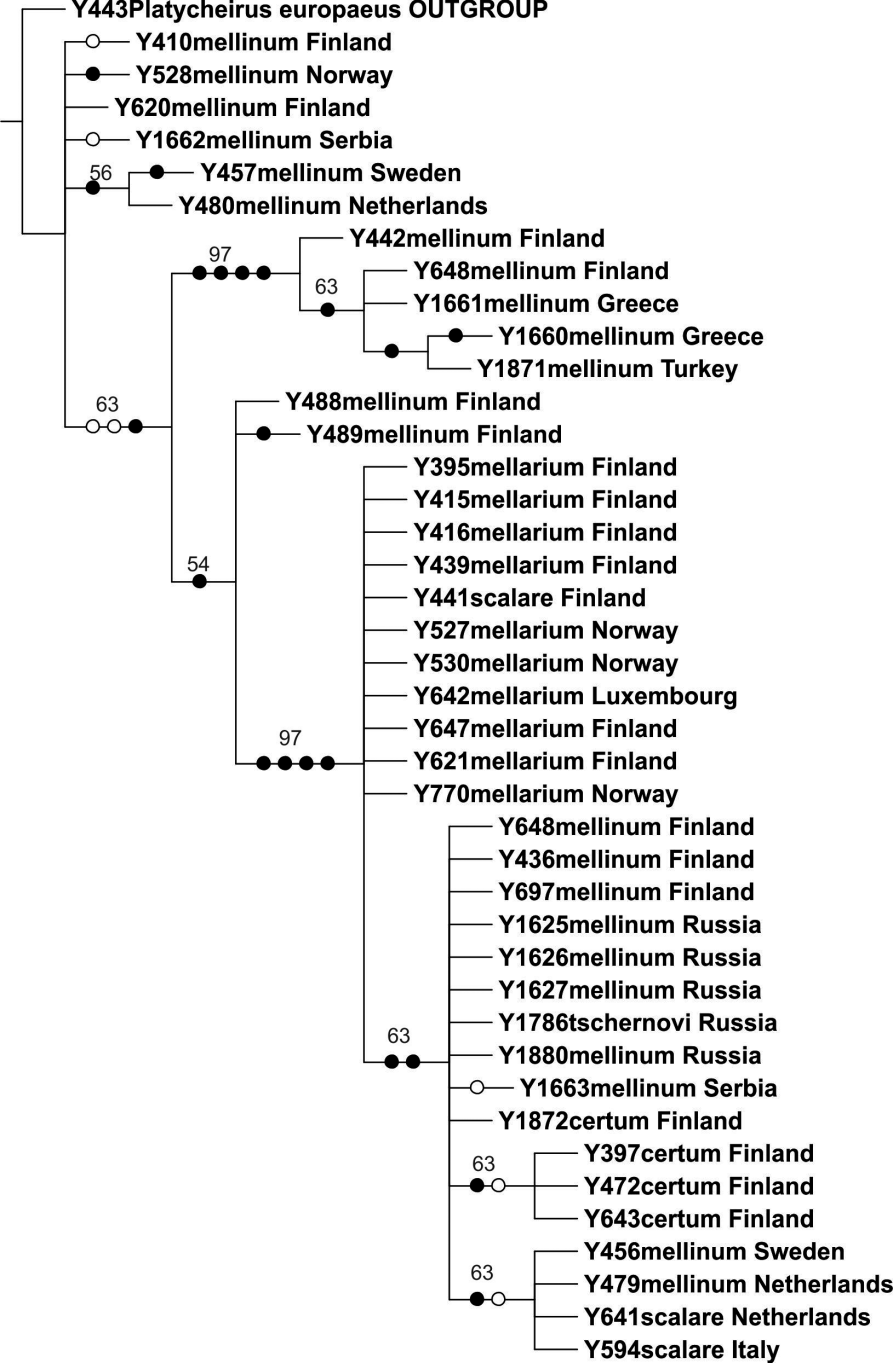
Strict consensus tree resulting from parsimony analysis of mtDNA COI gene. Filled circles denote unambiguous nucleotide changes, open circles ambiguous changes. Bootstrap support values indicated above branches.

The COI dataset comprised 18 parsimony informative characters. The parsimony analysis of the COI gene resulted in 72 equally parsimonious trees of 98 steps; the strict consensus tree is shown in Fig. 2 (taxa labelled according to new results). The COI gene 3'–fragment contained 18 variable sites (Table 2). We recorded two haplotypes for *Melanostoma certum* sp. n., one unique and one shared with *Melanostoma mellinum*, and 16 haplotypes for *Melanostoma mellinum* (in traditional sense) (*Melanostoma mellinum* specimens with uncorrected sequence divergence < 1%), one of which was shared with *Melanostoma certum* sp. n., and another one shared with *Melanostoma scalare* (Fig. 2, Table 2). *Melanostoma mellarium* had one haplotype which was shared with *Melanostoma scalare*, thus *Melanostoma scalare* showed no unique haplotypes for the COI gene region for the present dataset. All sequences of *Melanostoma tschernovi*, *Melanostoma dubium* and *Melanostoma mellinum* (no sequence obtained for *Melanostoma clausseni*) obtained from Russia clustered among *Melanostoma mellinum* samples.

**Figure 3.**
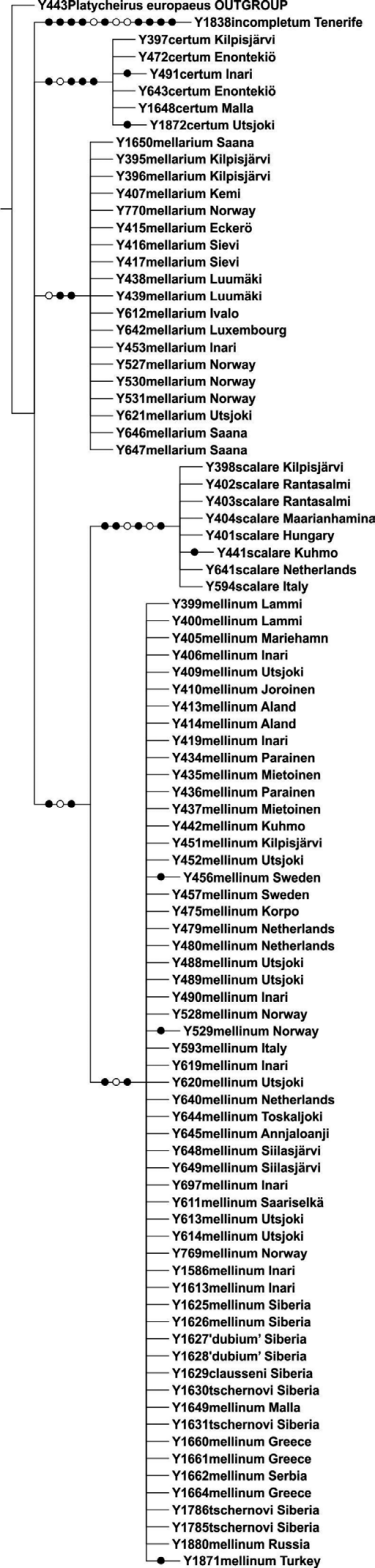
Strict consensus tree resulting from parsimony analysis of nuclear ITS2 gene region. Filled circles denote unambiguous nucleotide changes, open circles ambiguous changes. Samples from Finland labelled with locality names, from elsewhere with country name (for more information see Table 1). Bootstrap support values indicated above branches.

The parsimony analysis of the ITS2 marker resulted in two equally parsimonious trees of 155 steps, and the strict consensus tree is shown in Fig. 3. The ITS2 marker showed very low intraspecific variability (0.4%), and interspecific variability ranged between 2.6–6.0%. The ITS2 tree resolved the included *Melanostoma* specimens as five non-overlapping clades, with no samples exhibiting shared genotypes between the taxa. Again, all Russian samples (this time including *Melanostoma clausseni*) clustered within the *Melanostoma mellinum* clade (Fig. 3).

**Table 2.** MtDNA COI gene 3'–fragment, haplotype variation of 18 non-continuous sites among ingroup samples. Haplotypes are indicated with Roman numerals for each taxon. Samples listed by country, for Finnish sample localities North, Central or South Finland is indicated. The colors indicate haplotypes shared by two taxa.

Code	Taxon	020	050	071	110	140	206	272	281	284	332	419	434	521	548	578	635	699	737	Haplotype number	Locality
MZH_Y397	**certum**	T	A	T	T	T	C	A	T	G	T	A	C	C	-	-	-	-	-	I	Finland: N
MZH_Y472	**certum**	T	A	T	T	T	C	A	T	G	T	A	C	C	A	C	G	A	T	I	Finland: N
MZH_Y643	**certum**	T	A	T	T	T	C	A	T	G	T	A	C	C	A	C	G	A	T	I	Finland: N
MZH_Y1872	**certum**	T	A	T	T	T	C	A	T	G	T	A	T	C	A	C	G	G	T	II	Finland: N
MZH_Y648	**mellinum**	T	A	A	C	A	C	A	C	A	G	G	T	C	A	T	A	A	C	I	Finland: N
MZH_E62	**mellinum**	T	A	A	C	A	T	A	C	A	G	G	T	C	A	T	A	A	C	II	Cyprus
MZH_Y442	**mellinum**	T	A	A	C	A	T	A	C	A	G	G	T	C	A	T	A	A	T	III	Finland: C
S564	**mellinum**	T	A	A	C	A	T	A	C	A	T	G	T	C	A	T	A	A	T	IV	Netherlands
MZH_Y1871	**mellinum**	T	A	A	C	A	T	A	C	A	A	G	T	C	A	T	A	A	C	V	Turkey
MZH_Y1660	**mellinum**	T	A	A	T	A	T	A	C	A	A	G	T	C	A	T	G	A	C	VI	Greece
MZH_Y1661	**mellinum**	T	A	A	T	A	T	A	C	A	G	G	T	C	A	T	G	A	C	VII	Greece
MZH_Y410	**mellinum**	C	A	A	T	A	T	A	T	A	T	A	T	T	A	T	A	A	T	VIII	Finland: C
MZH_Y419	**mellinum**	C	G	A	T	A	T	A	T	A	T	A	T	T	A	T	A	A	T	IX	Finland: N
MZH_Y528	**mellinum**	C	G	A	T	A	T	A	T	A	T	A	T	T	A	T	A	A	T	IX	Norway
MZH_Y457	**mellinum**	C	G	A	T	A	T	A	T	A	T	A	T	T	G	T	A	A	T	X	Sweden
MZH_Y480	**mellinum**	C	G	A	T	A	T	A	T	A	T	A	T	T	G	T	A	A	T	X	Netherlands
MZH_Y488	**mellinum**	T	A	A	T	A	T	A	T	A	T	A	T	C	A	T	G	A	T	XI	Finland: N
MZH_Y489	**mellinum**	T	A	A	T	A	T	A	T	A	T	A	T	C	A	T	G	A	T	XI	Finland: N
MZH_Y697	**mellinum**	T	A	T	T	T	C	A	T	G	T	A	T	C	A	C	G	G	T	XII	Finland: N
MZH_Y436	**mellinum**	T	A	T	T	T	C	A	T	G	T	A	T	C	A	C	G	G	T	XII	Finland: S
MZH_Y437	**mellinum**	T	A	T	T	T	C	A	T	G	T	A	T	C	A	C	G	G	T	XII	Finland: S
MZH_Y1625	**mellinum**	T	A	T	T	T	C	A	T	G	T	A	T	C	A	C	G	n	T	XII	Russia
MZH_Y1626	**mellinum**	T	A	T	T	T	C	A	T	G	T	A	T	C	A	C	G	n	T	XII	Russia
MZH_Y1627	’**dubium**’	T	A	T	T	T	C	A	T	G	T	A	T	C	A	C	G	n	T	XII	Russia
MZH_Y1880	**mellinum**	T	A	T	T	T	C	A	T	G	T	A	T	C	A	C	G	G	T	XII	Russia
MZH_Y1786	’**tschernovi**’	T	A	T	T	T	C	A	T	G	T	A	T	C	A	C	G	G	T	XII	Russia
MZH_Y1880	**mellinum**	T	A	T	T	T	C	A	T	G	T	A	T	C	A	C	G	G	T	XII	Russia
MZH_Y456	**mellinum**	T	A	T	T	T	C	G	T	G	T	A	T	C	A	C	G	A	T	XIII	Sweden
MZH_Y479	**mellinum**	T	A	T	T	T	C	G	T	G	T	A	T	C	A	C	G	A	T	XIII	Netherlands
MZH_Y594	**scalare**	T	A	T	T	T	C	G	T	G	T	A	T	C	A	C	G	A	T	XIII	Italy, Sard.
MZH_Y641	**scalare**	T	A	T	T	T	C	G	T	G	T	A	T	C	A	C	G	A	T	XIII	Netherlands
MZH_Y1663	**mellinum**	T	A	T	C	T	C	A	T	G	T	A	T	C	A	C	G	A	T	XIII	Serbia
MZH_Y1662	**mellinum**	T	G	A	C	A	T	A	T	A	T	A	T	T	A	T	G	A	T	XIV	Serbia
MZH_E61	**mellinum**	T	A	T	T	A	T	G	T	G	T	A	T	C	A	C	A	A	T	XV	Cyprus
MZH_Y395	**mellarium**	T	A	T	T	T	C	A	T	A	T	A	T	C	A	C	G	A	T	I	Finland: N
MZH_Y415	**mellarium**	T	A	T	T	T	C	A	T	A	T	A	T	C	A	C	G	A	T	I	Finland: S
MZH_Y416	**mellarium**	T	A	T	T	T	C	A	T	A	T	A	T	C	A	C	G	A	T	I	Finland: C
MZH_Y439	**mellarium**	T	A	T	T	T	C	A	T	A	T	A	T	C	A	C	G	A	T	I	Finland: C
MZH_Y527	**mellarium**	T	A	T	T	T	C	A	T	A	T	A	T	C	A	C	G	A	T	I	Norway
MZH_Y530	**mellarium**	T	A	T	T	T	C	A	T	A	T	A	T	C	A	C	G	A	T	I	Norway
MZH_Y770	**mellarium**	T	A	T	T	T	C	A	T	A	T	A	T	C	A	C	G	A	T	I	Norway
MZH_Y441	**scalare**	T	A	T	T	T	C	A	T	A	T	A	T	C	A	C	G	A	T	I	Finland: C

#### Melanostoma

Taxon classificationAnimaliaDipteraSyrphidae

Schiner, 1860

##### Description.

The description is based on Vockeroth (1992) and on own findings.

*Head*: Eyes bare. Frontal triangle of male blackish, shining or with variable amount of dusting. Frons of female blackish, mostly shining with a pair of triangular dusted maculae above lunule. The size of these maculae varies and they are medially separated or confluent. Face and shallow facial tubercle blackish, shining or with a variable amount of dusting. Lunule black and shiny. Antenna varying from totally dark brown to largely yellow with brown dorsal margin of basoflagellomere.

*Thorax*: Scutum blackish, shining, usually with slight dusting anteriorly and laterally. Pili on scutum predominantly yellowish or whitish, rarely partly or totally blackish. Scutellum shining. Pleura mostly bare, blackish, shining or with variable amount of dusting. Katepimeron with widely separated dorsal and ventral pile patches. Metasternum consists of only a narrowly sclerotized anterior and median stripe. *Wing*: Usually totally microtrichose, at most with small bare areas around base of cell BM.

*Legs*: Coxa blackish. Metacoxa without posterior pile tuft. Femur, tibia and tarsus slender without outstanding pile or bristles. Coloration varies from almost totally yellow to almost totally dark brown.

*Male abdomen*: Nearly parallel sided, two to five times as long as greatest width. Terga 2–4 usually with sub-rectangular yellow maculae, but maculae sometimes darkened and/or reduced to various extent. Yellow maculae shining or with various amount of dusting. Maculae on tergum 2 separated from anterior margin. Maculae on terga 3–4 usually reaching anterior margins. Maculae on terga 2–4 usually reaching lateral margins and separated from posterior margins.

*Female abdomen*: Shape varying from nearly parallel sided to oval; two to four times as long as its greatest width. Terga 2–5 usually with yellow maculae but these maculae sometimes darkened and/or reduced to some extent. Maculae on tergum 2 roundish and separated from the margins. Terga 3–4 with anterior triangular maculae narrowly reaching lateral margins. Tergum 5 with or without anterolateral maculae.

**Figure 4.**
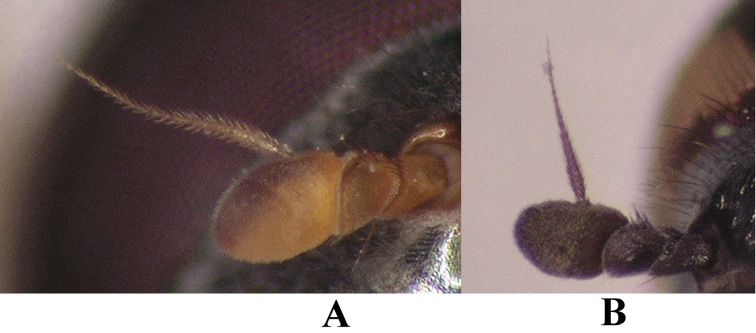
Antenna. **A** *Melanostoma scalare*, male and **B** *Melanostoma certum*, male.

**Figure 5.**
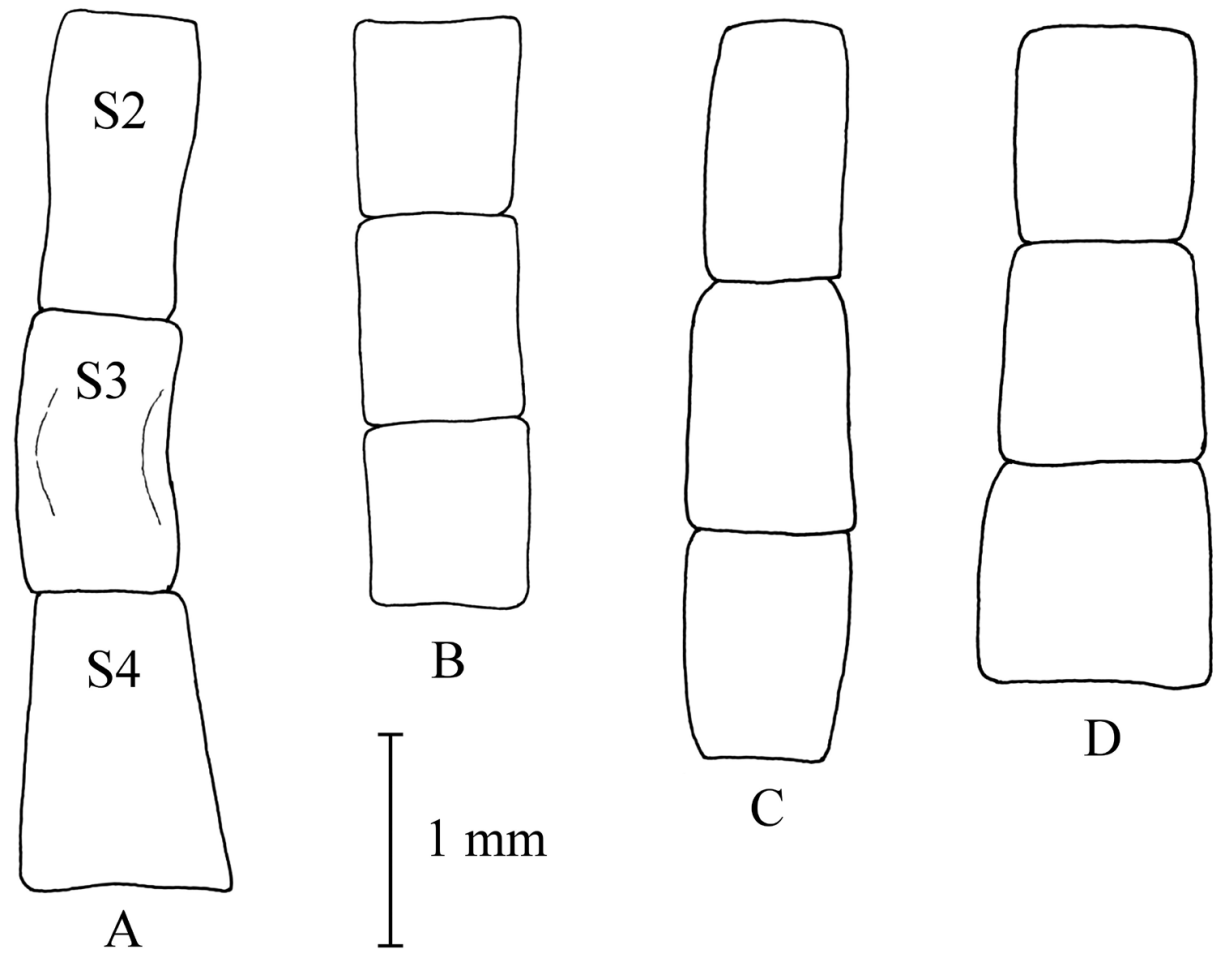
Shape of male sterna 2–4. **A** *Melanostoma scalare* **B** *Melanostoma certum* **C** *Melanostoma mellarium* and **D** *Melanostoma mellinum*.

**Figure 6.**
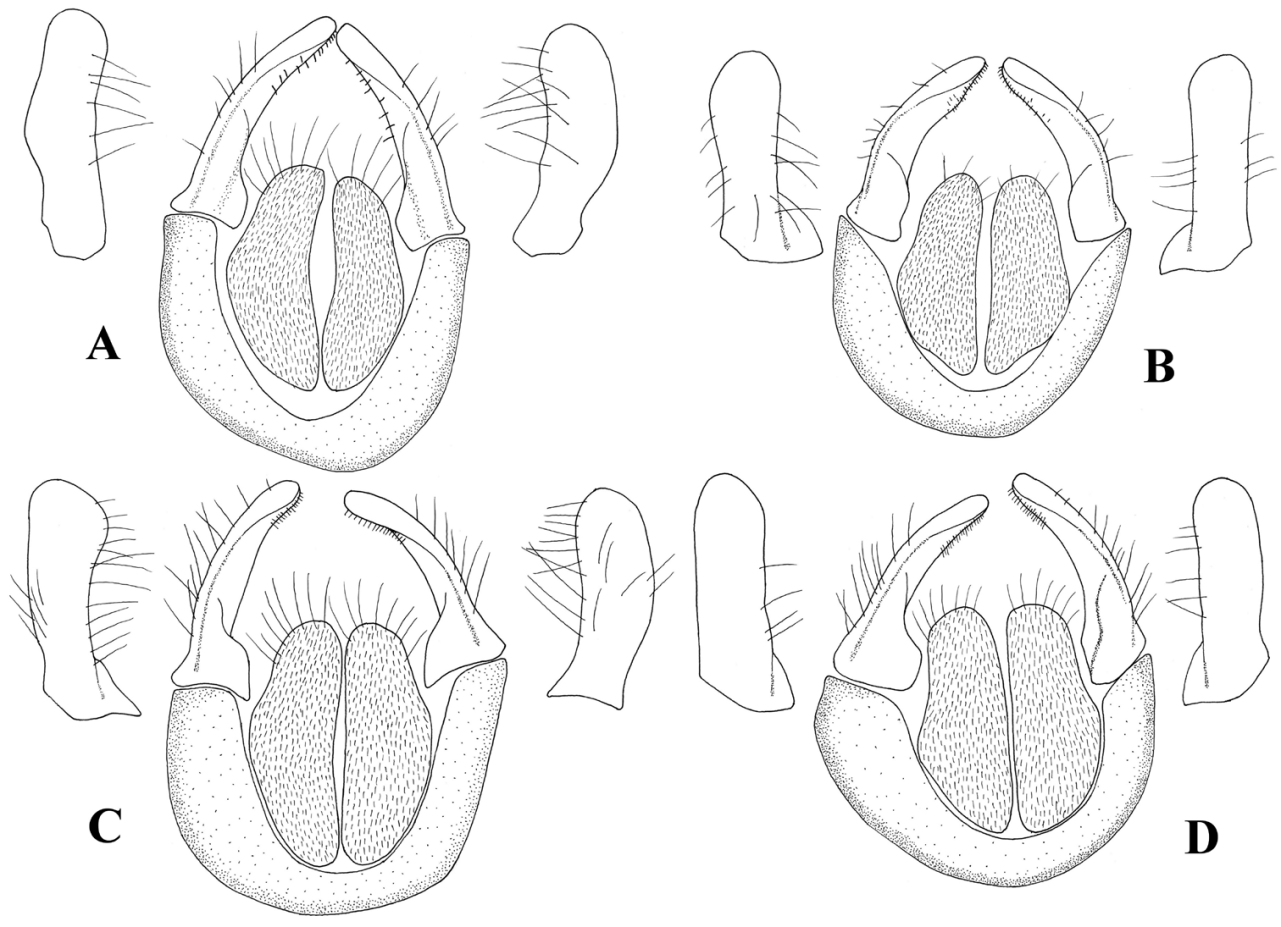
Cerci and surstyli, dorsal view and surstyli, lateral view. **A** *Melanostoma scalare* **B** *Melanostoma certum* **C** *Melanostoma mellarium* and **D** *Melanostoma mellinum*.

**Figure 7.**
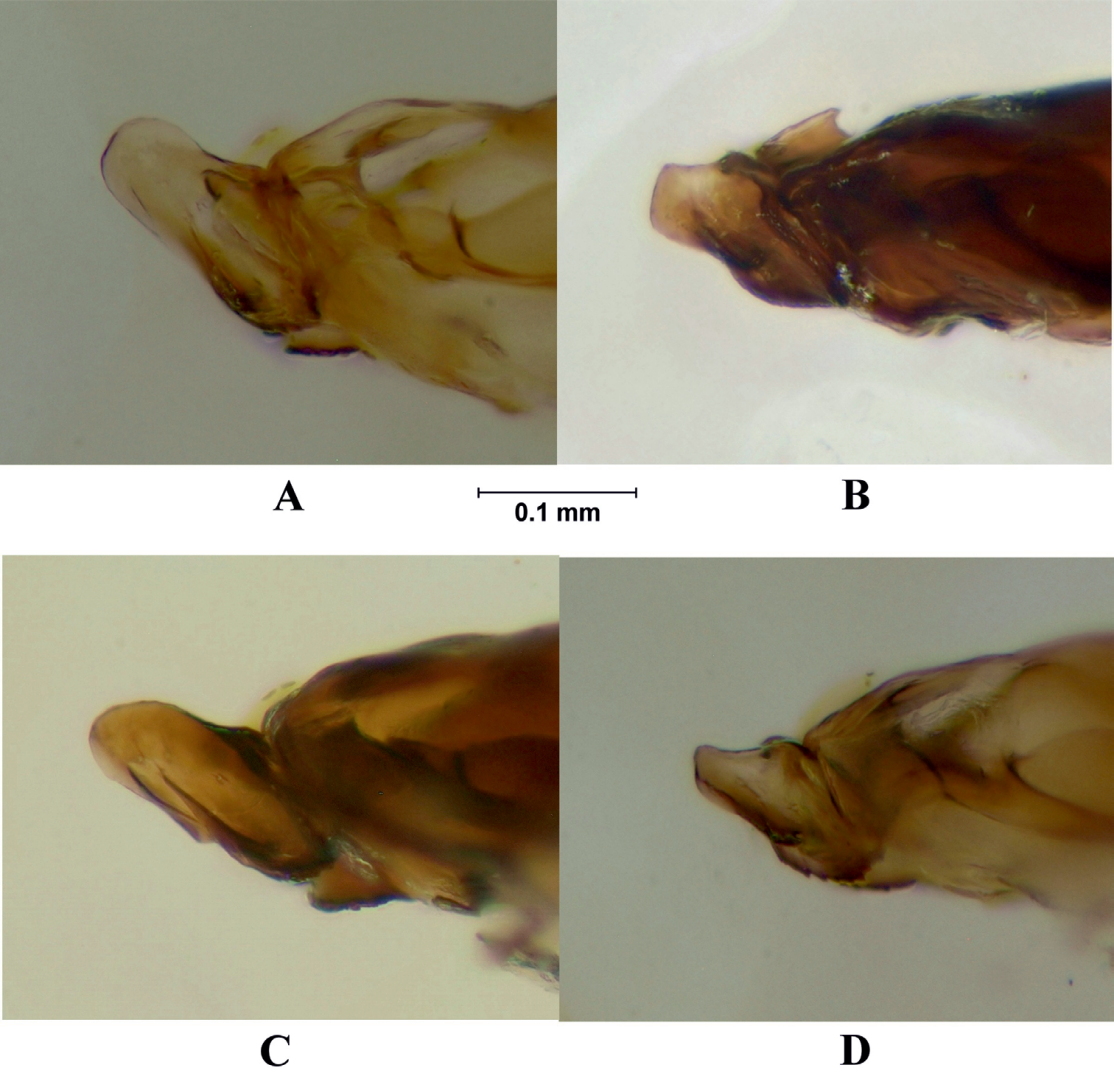
Postgonite and anterior part of hypandrium, lateral view. **A** *Melanostoma scalare* **B** *Melanostoma certum* **C** *Melanostoma mellarium* and **D** *Melanostoma mellinum*.

#### Melanostoma certum sp. n.

Taxon classificationAnimaliaDipteraSyrphidae

http://zoobank.org/843A2625-9859-4486-9FEA-04865F72F4CE

Figs 4B , 5B , 6B , 7B , 8B , 9B , 10B , 11B , 12B , 12C , 14B , 15B , 16B

Melanstoma dubium auctt. nec Zetterstedt, 1838

##### Type-locality.

FINLAND: Le: Enontekiö, Annjaloanji, [69°10'9"N, 21°25'22"E], ykj76865:2790.

##### Types.

**Holotype:** male, pinned, deposited in MZH. Original labels: ‘Finland, [69°10'9"N, 21°25'22"E], ykj76865:2790, Le: Enontekiö, Annjaloanji, 13.7.2007, A. Haarto leg. / DNA voucher specimen MZH_Y643, G. Ståhls, FMNH, Helsinki, Finland / Holotype *Melanostoma certum* Haarto & Ståhls 2013’. **Paratypes:** 1 male, FINLAND: EnL: Enontekiö Korkea Jehkas lampi, [69°4'39"N, 20°50'58"E], ykj76785:2553, 20.7.2005, K. Mattila leg. / Paratype *Melanostoma certum* Haarto & Ståhls 2013’, [AHPC]; 1 male, FINLAND: Li: Utsjoki Pulmanki, [70°2'26"N, 27°53'53"E], ykj 77739:5344, 5.7.2004, J. Kahanpää leg. / Paratype *Melanostoma certum* Haarto & Ståhls 2013’, [MZT]; 1 female, FINLAND: Li: Utsjoki, Karigasniemi, Ailigas, [69°24'51"N, 25°58'45"E], ykj77041:4601, 6.VII.2004, J. Kahanpää leg. / *Melanostoma dubium* A. Haarto det. / Paratype *Melanostoma certum* Haarto & Ståhls 2013’, [MZH]; 1 female, FINLAND: Le: Enontekiö Annjaloanji, [69°10'9"N, 21°25'39"E], ykj76865:2790, 13.7.2007 (puro), A. Haarto leg. / Paratype *Melanostoma certum* Haarto & Ståhls 2013’, [AHPC]; 1 female, FINLAND: Le: Enontekiö Jogasjärvi, [69°9'58"N, 21°27'50"E], ykj76860:2806, 11–16.7.2007, malaise, R. Jussila leg. / Paratype *Melanostoma certum* Haarto & Ståhls 2013’, [MZT]; 1 female FINLAND: Le: Enontekiö Bumbovarri, [69°11'N 21 29'E], ykj7686:328, 9.7.2007, J.-P. Kaitila & M. Rantala leg. / Paratype *Melanostoma certum* Haarto & Ståhls 2013’, [SKPC]; 1 female FINLAND: Le: Enontekiö Annjaloanji, [69°10'N, 21°26'E], ykj7686:328, 12.7.2007, J.-P. Kaitila & M. Rantala leg. / Paratype *Melanostoma certum* Haarto & Ståhls 2013’, [SKPC]; 1 female FINLAND: Le: Enontekiö Toskaljoki, [69°10'34"N, 21°27'34"E], ykj76871:328, 12.7.2008, J.-P. Kaitila & M. Rantala leg. / Paratype *Melanostoma certum* Haarto & Ståhls 2013’, [SKPC]; 1 male FINLAND: Le: Enontekiö Toskaljoki, [69°10'34''N, 21°27'34''E], 76871:328, 11.7.2008, J.-P. Kaitila & M. Rantala leg. / Paratype *Melanostoma certum* Haarto & Ståhls 2013’, [SKPC]; and six DNA voucher paratype specimens in MZH as listed in Table 1.

##### Male.

*Head*: Colour greyish black. Angle of approximation of eyes 85°–100°. Eye contiguity about as long as frontal triangle. Ocellar triangle slightly longer than wide with dark pile and indistinctly grey dusted. Occiput very narrow and usually with pale pile. Frontal triangle with indistinct or thin grey dusting. Lateral parts of frontal triangle with dark pile. Face shining with indistinct or thin grey dusting. Lateral parts of face with dark and pale pile. Gena about as wide as basoflagellomere and with greyish dusting. Antenna dark brown. Basoflagellomere about 1.3 times as long as wide. Arista brown and almost twice as long as basoflagellomere. Arista with extremely short pile, longest pile at the most half width of arista at base (Fig. 4B).

*Thorax*: Scutum shining greyish black except for thin greyish dusting at anterior margin. Scutum with erect whitish pile and with shorter semi-adpressed whitish pile on anterior margin. Anterior part of scutum with long erect pile which length at least third of length of scutellum. Postpronotum totally covered by thin greyish dusting. Notopleuron with thin greyish dusting. Scutellum shining greyish black, with whitish erect pile at its dorsum, hind margin and ventral side. Pleura greyish black with thin greyish dusting. Pleura with pale erect pile. Calypter brownish with pale brownish pile at edge. Halter yellow, with slightly darkened base of stem. *Wing*: Completely microtrichose, with slightly brownish ting. Stigma yellowish brown.

*Legs*: Coxa black with grey dusting. Trochanter dark brown. Femur usually mainly black except narrowly yellow apically. Tibia usually mainly dark brown except narrowly yellow bases and apices. Tarsus usually dark brown. Leg with pale and dark pile mixed.

**Figure 8.**
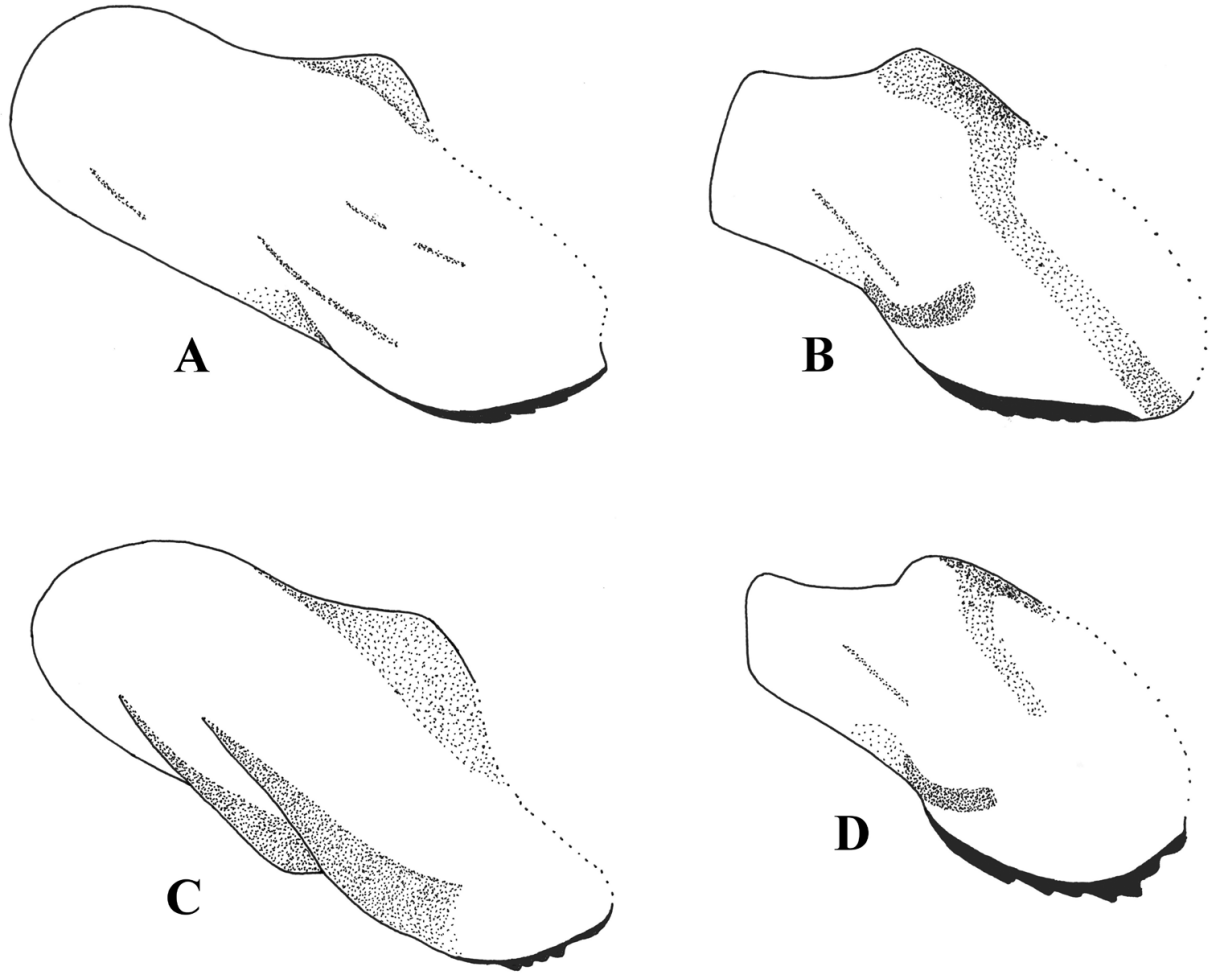
Postgonite, lateral view. **A** *Melanostoma scalare* **B** *Melanostoma certum* **C** *Melanostoma mellarium* and **D** *Melanostoma mellinum*.

**Figure 9.**
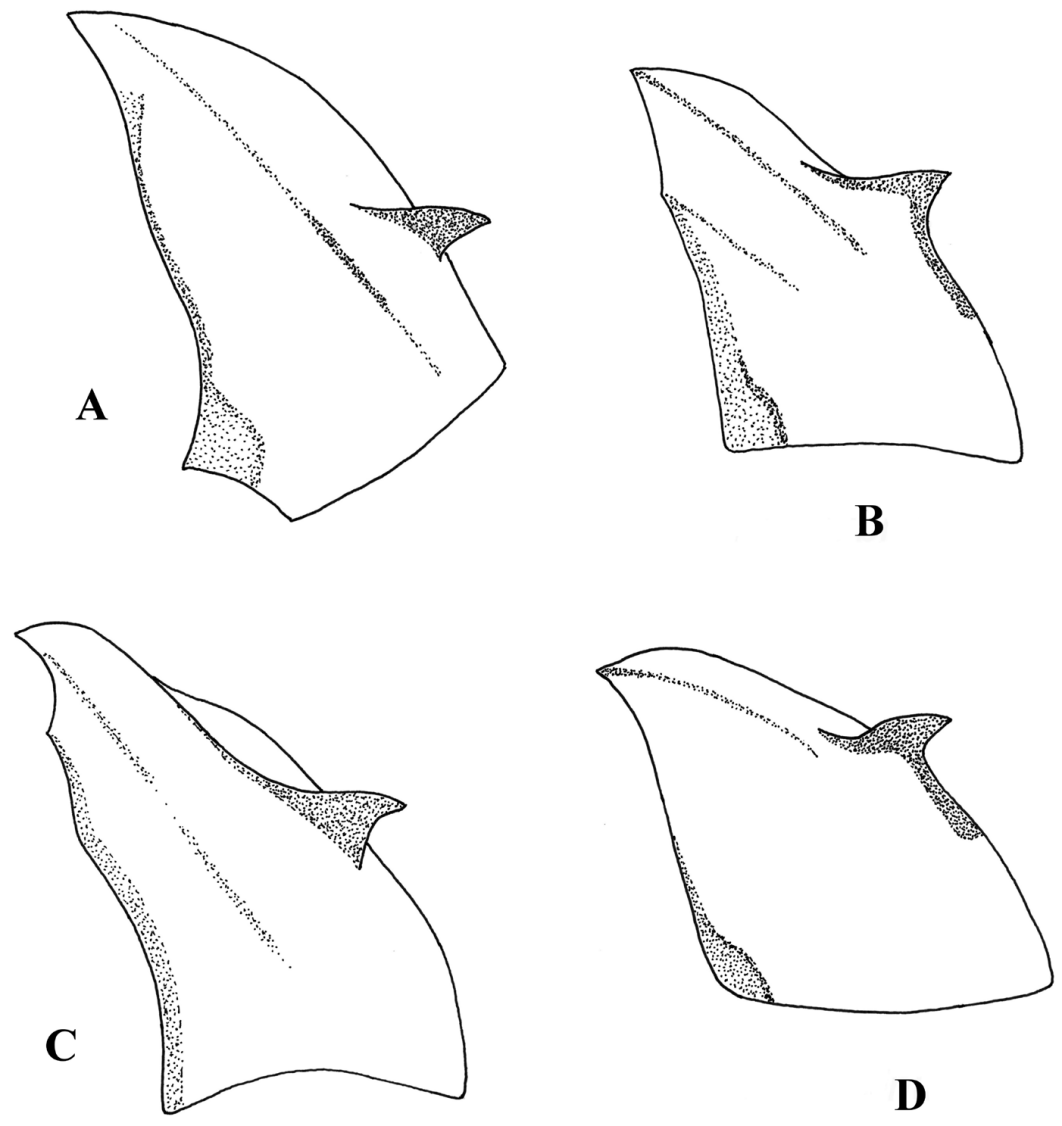
Postgonite, ventral view. **A** *Melanostoma scalare* **B** *Melanostoma certum* **C** *Melanostoma mellarium* and **D** *Melanostoma mellinum*.

**Figure 10.**
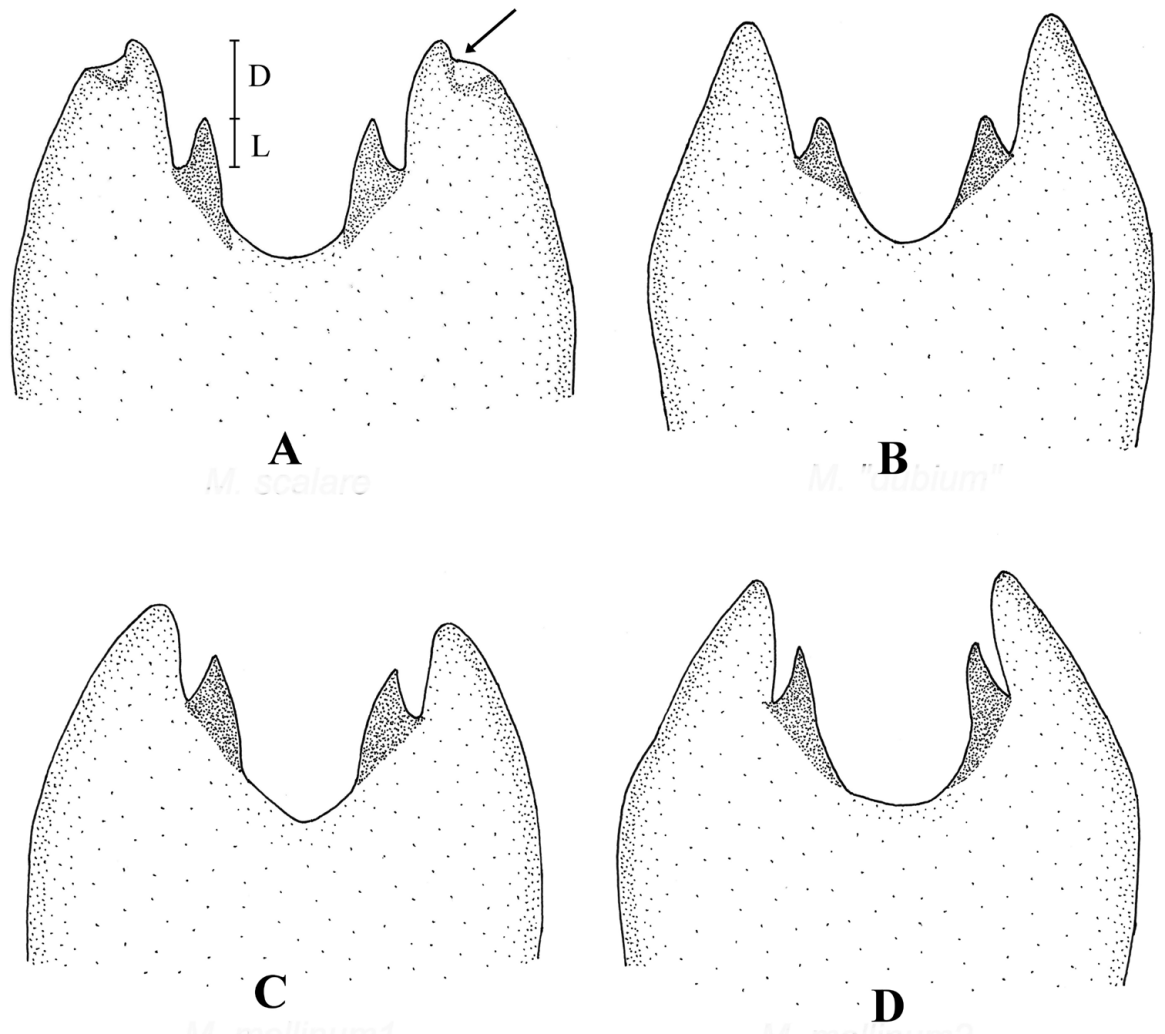
Hypandrium, lateral view. **A** *Melanostoma scalare* **B** *Melanostoma certum* **C** *Melanostoma mellarium* and **D** *Melanostoma mellinum*.

**Figure 11.**
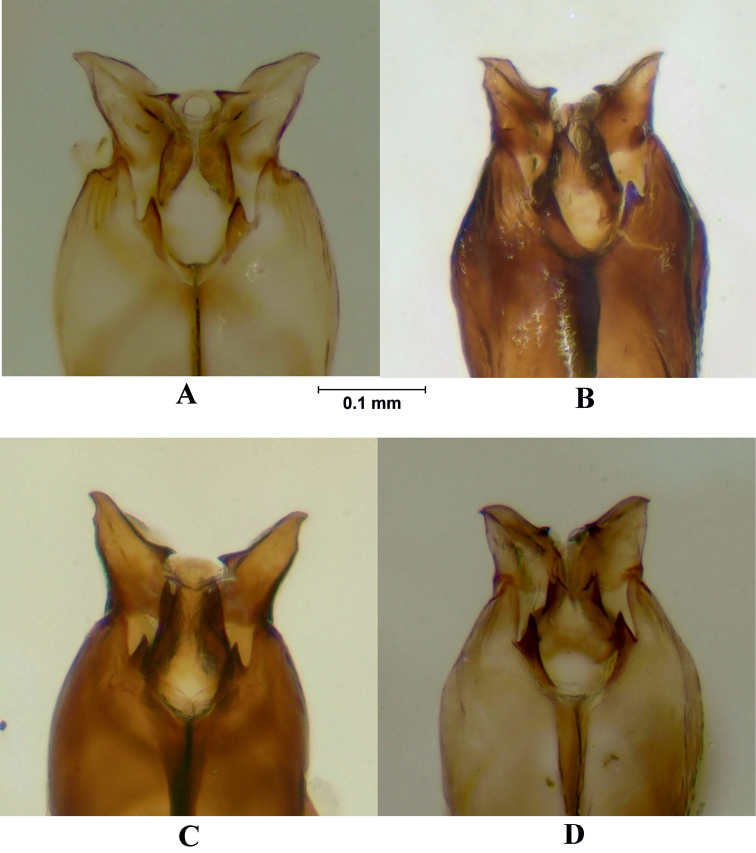
Hypandrium, ventral view, shape of postgonites **A** *Melanostoma scalare* **B** *Melanostoma certum* **C** *Melanostoma mellarium* and **D** *Melanostoma mellinum*.

*Abdomen*: Terga dark brown or black with dense or weak brownish dusting. Tergum 2 without or with pair of yellow roundish maculae. Terga 3 and 4 with pair of yellow subrectangular maculae. Terga 1 and 2 laterally with long whitish pile. Terga with pale and usually some dark semi-adpressed pile outside of yellow maculae. Only pale pile on yellow maculae. Terga 2, 3 and 4, each about 1.2 times as long as its width. Sterna weakly dusted and with pale semi-adpressed pile. Sternum 2 about 1.4 times as long as wide at its posterior margin. Sternum 3 about 1.3 times as long as wide at its anterior margin. Sternum 4 about 1.3 times as long as wide at its anterior margin. Shape of sterna 2–4 is shown in Fig. 5B. *Male genitalia*: Cercus and surstylus as in Fig. 6B. Postgonite short and without distinct ridges laterally (Figs 7B, 8B). Postgonite ventrally as in Fig. 9B. Margin of hypandrium near postgonites with short triangular projections, index DL more than 2.2 (Figs 10B, 11B).

##### Female.

Similar to male, but differs as follows:

*Head*: Frons shining except greyish dusted triangles which usually are medially confluent. Ventral to the dusted triangles the thinly dusted area is usually reaching the lateral area of lunule. Frons at level of front ocellus slightly narrower than length of antenna. Dorsal part of frons with dark pile and ventral part of frons with pale pile. Occiput as broad as two diameters of an ocellus and usually with pale pile.

*Thorax*: Scutum and scutellum with short pale pile. Calypter pale brownish with whitish pile at edge.

*Legs*: Coloration similar to male.

**Figure 12.**
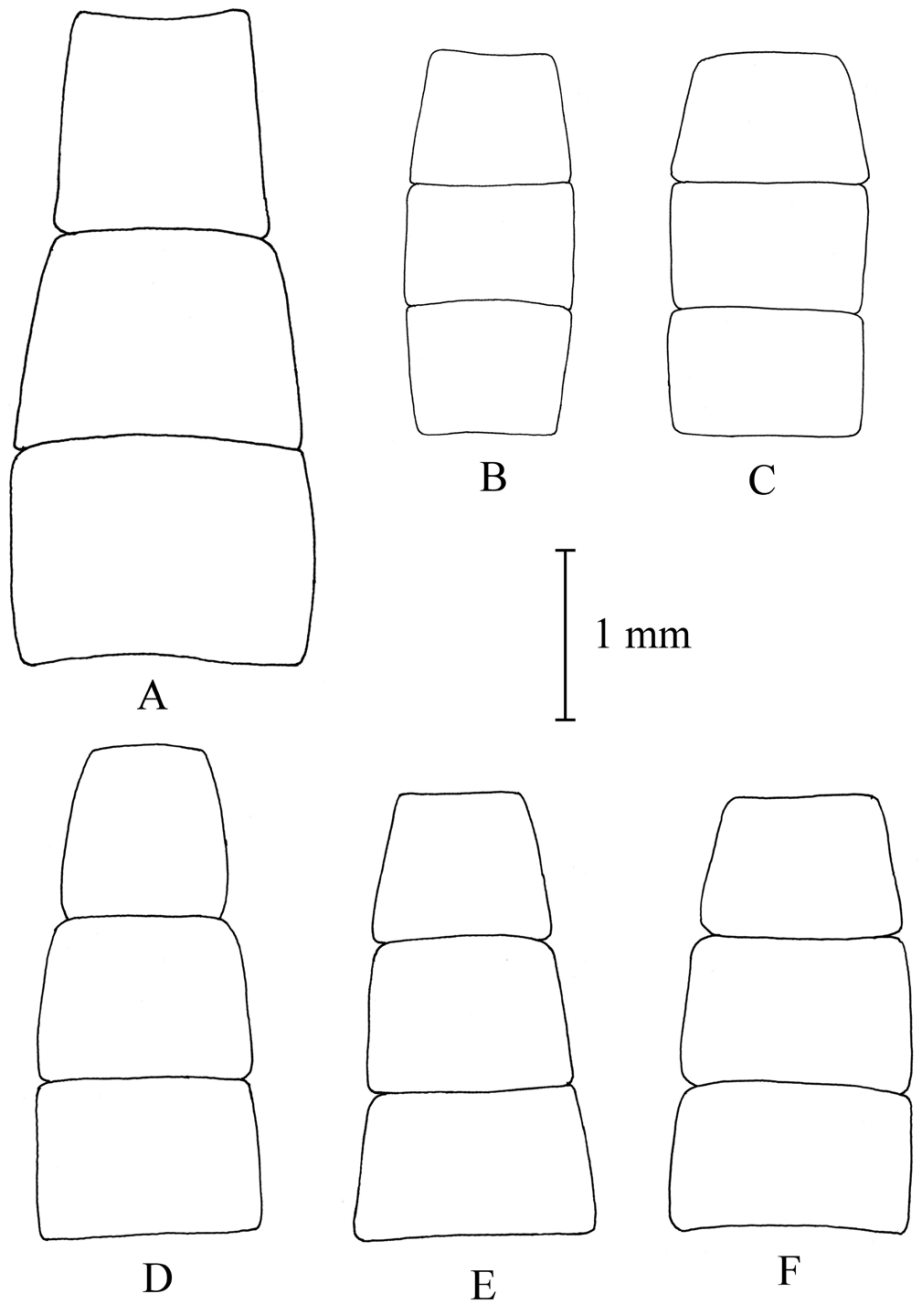
Shape of female sterna 2–4. **A** *Melanostoma scalare*, **B** and **C** *Melanostoma certum* **D** *Melanostoma mellarium* and **E** and **F** *Melanostoma mellinum*.

*Abdomen*: Terga 1 and 2 laterally with long whitish pile. Terga 2–5 dorsally with whitish semi-adpressed pile and always without yellow maculae. Terga 2 and 3 each about 0.5 times as long as wide at its posterior margin. Tergum 4 about 0.6 times as long as wide at its posterior margin. Sternum 2 about 0.6 times as long as wide at its posterior margin. Sternum 3 about 0.6 times as long as wide at its anterior margin. Sternum 4 at least 0.7 times as long as wide at its anterior margin. Sterna 3 and 4 almost parallel sided, rarely slightly broadened towards posterior margins. Shape of sterna 2–4 is shown in Fig. 12B, C.

##### Length

(4 males and 6 females): Body 5–7 mm.

##### Distribution.

All verified specimens originate from North European localities north of 68°N, and almost all specimens are sampled at or above the tree line.

##### Etymology.

The word *certum* means clear, defined, and is to be treated as adjective in neutrum.

#### Melanostoma mellarium

Taxon classificationAnimaliaDipteraSyrphidae

(Meigen, 1822) stat. n.

Figs 5C , 6C , 7C , 8C , 9C , 10C , 11C , 12D , 13A , 14C

Syrphus mellarius Meigen, 1822.
Melanostoma mellinum var. *melanatus* Kanervo, 1934, syn. n.

##### Type-locality.

The locality of the lectotype is not indicated in the original label. Peck (1988) gave the following information “Auf Wiesen und in Wäldern nicht selten''“?Stolberg near Aachen''[Germany].

##### Types.

**Lectotype of *Melanostoma mellarium*:** female, pinned, deposited in MNHN. Original label: ’Meigen 1486 40 / mellinum type’. Here the lectotype is designated to fix the concept of *Melanostoma mellarium* (Meigen) and to ensure the universal and consistent interpretation of the same. Labelled: ‘LECTOTYPE *Melanostoma mellarium* (Meigen, 1822), Haarto & Ståhls des. 2013’. Images from MNHN.

##### Lectotype

**of *Melanostoma mellinum* var. *melanatus*:** female, pinned, deposited in MZT. Original label: ‘Haukilampi, 28.4.28'‘Lectotype *Melanostoma mellinum* var. *melanatus* Kanervo, Haarto & Ståhls des. 2014’.

##### Additional material studied.

DNA voucher specimens in MZH (Table 1) 17 males and 30 females in MZH; 25 male and 25 female specimens in AHPC.

##### Male.

*Head*: Colour black. Angle of approximation of eyes 80°–90°. Eye contiguity about as long as frontal triangle. Ocellar triangle slightly longer than wide with dark pile and with indistinct grey dusting. Occiput very narrow and dorsally with dark pile and laterally with pale pile. Frontal triangle shining with indistinct grey dusting. Lateral parts of frontal triangle with dark pile. Face shining with indistinct grey dusting. Lateral parts of face with pale and dark pile. Gena about as wide as basoflagellomere and with thin greyish dusting. Antenna mainly dark brown, basoflagellomere usually with a yellow spot baso-ventrally. Basoflagellomere about 1.3 times as long as its width. Arista usually brown and about twice as long as basoflagellomere. Longest pile of arista at most half width of arista at base as in Fig. 4B.

*Thorax*: Scutum shining black except for thin greyish dusting at anterior margin. Scutum usually with pale brown and dark erect pile and with shorter semi-adpressed pale pile on anterior margin. Pile rarely mainly dark on scutum. Anterior part of scutum with short erect pile which length about fourth part of length of scutellum. Postpronotum totally covered by thin greyish dusting. Notopleuron covered by indistinct greyish dusting. Scutellum shining black with pale and dark erect pile at its dorsum and posterior margin. Scutellum only pale pile at its ventral side. Pleura black and usually with only thin grey dusting and usually more distinctly shining on posterior part of anepisternum, anterior part of anepimeron and dorsal part of katepisternum. Pleura with pale erect pile. Calypter brownish with pale brownish pile on margin. Halter yellow with slightly darkened base of stem. *Wing*: Usually completely microtrichose, rarely with small bare area on base of cell BM. Membrane with slightly brownish ting. Stigma yellowish brown.

*Legs*: Coxa black with grey dusting. Trochanter dark brown. Femur usually mainly black except yellow apical part. Tibia usually mainly yellow with dark brown ring varying size. Metatibia usually with a longer dark ring than other tibiae. Tarsus dark brown except mesotarsus with two basal segments yellow. Leg with pale and dark pile mixed.

*Abdomen*: Terga dark brown or black with weak greyish dusting. Tergum 2 with pair of yellow oval maculae. Terga 3 and 4 with pair of yellow subrectangular maculae. Terga 1 and 2 laterally with long pale pile. Terga with varying amount of dark and pale semi-adpressed pile outside of yellow maculae. Only pale pile on yellow maculae. Terga 2, 3 and 4 each about 1.4 times as long as its width. Sterna with weak dusting and with pale semi-adpressed pile. Sternum 2 about 1.6 times as long as its width at its posterior margin. Sternum 3 about 1.5 times as long as its width at its anterior margin. Sternum 4 about 1.4 times as long as its width at its anterior margin. Shape of sterna 2–4 are shown in Fig. 5C. *Male genitalia*: Cercus and surstylus as in Fig. 6C. Postgonite long and with distinct ridges laterally (Figs 7C, 8C). Postgonite ventrally in Fig. 9C. The hypandrial, margin at postgonites with long triangular projections, index DL less than 1.2 (Figs 10C, 11C).

##### Female.

Similar to male, but differs as follows:

*Head*: Frons shining except greyish dusted triangles. Frons at level of front ocellus slightly narrower than length of antenna. Dorsal part of frons with dark pile and ventral part of frons with pale pile. Occipital orbit as broad as two diameters of an ocellus and usually dorsally with pale and dark pile and laterally with pale pile. Scape and pedicel brown or yellowish brown.

*Thorax*: Scutum and scutellum with short pale pile. Calypter whitish yellow with whitish pile at edge. *Wing*: Indistinctly brownish tinged. Stigma pale yellowish brown.

*Legs*: Femur and tibia usually mainly yellow with dark brown ring varying size. Metaleg usually largely darker than other leg.

*Abdomen*: Terga indistinctly grey dusted. Tergum 2 without or with a pair of small yellow oval maculae. Terga 3 and 4 usually with a pair of small yellow elongated triangular maculae. Tergum 5 at anterior margin without or with pair of short yellow maculae. Tergum 2 about 0.6 times as long as its width at its posterior margin. Tergum 3 about 0.7 times as long as its width at its posterior margin. Tergum 4 about 0.9 times as long as its width at its posterior margin. Sternum 2 about 0.8 times as long as its width at its posterior margin. Sternum 3 about 0.8 times as long as its width at its anterior margin. Sternum 4 about 0.8 times as long as its width at its anterior margin. Shape of sterna 2–4 are shown in Fig. 12D.

##### Length

(25 males and 25 females): Body 7–9 mm.

##### Distribution.

We have verified specimens from Fennoscandia and central Europe, but data for a more detailed distributional map is presently not available.

**Figure 13.**
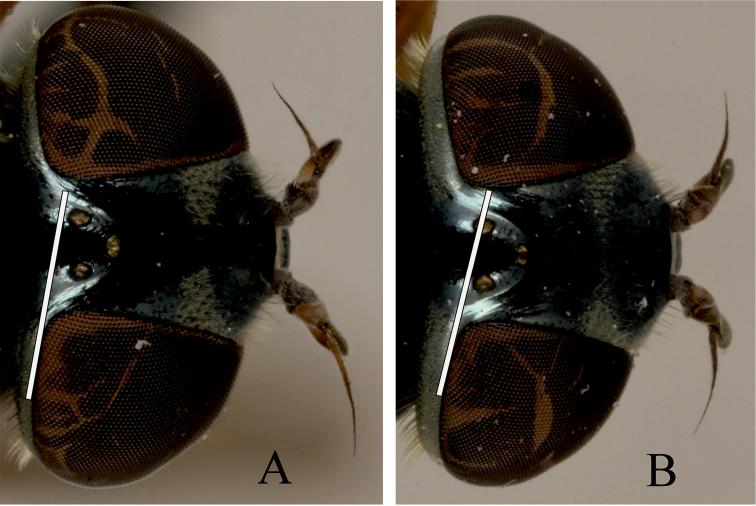
Dorsal view of female head. Position of posterior ocellus as compared to the hind eye line of female. **A** *Melanostoma mellarium* and **B** *Melanostoma mellinum*.

**Figure 14.**
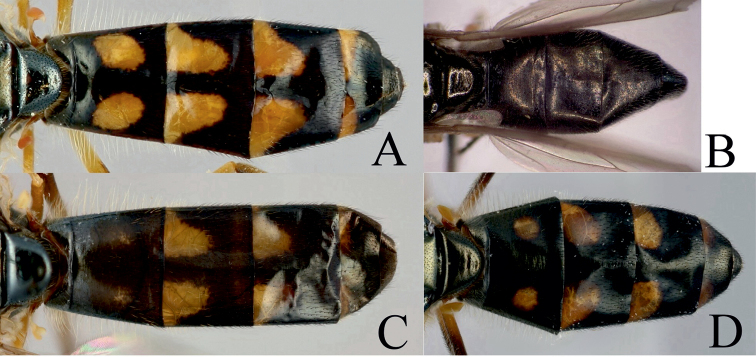
Abdomen of female. **A** *Melanostoma scalare* **B** *Melanostoma certum* **C** *Melanostoma mellarium* **D** *Melanostoma mellinum*.

#### Melanostoma mellinum

Taxon classificationAnimaliaDipteraSyrphidae

(Linnaeus, 1758)

Figs 5D 6D 7D 8D 9D 10D 11D 12E 12F 13B 14D 15A 16A

Musca mellina Linnaeus, 1758
Musca facultas Harris, 1780
Syrphus melliturgum Meigen, 1822
Syrphus minutum Macquart, 1829
Syrphus unicolor Macquart, 1829
Syrphus laevigatus Meigen, 1838
Syrphus concolor Walker, 1851
Melanostoma nigricorne Strobl, 1893
Melanostoma inormatum Matsumura, 1919
Melanostoma ochiaianum Matsumura, 1919
Melanostoma ogasawarae Matsumura, 1919
Melanostoma sachalinense Matsumura, 1919
Melanostoma deficiens Szilády, 1940
Melanostoma dilatatum Szilády, 1940
Scaeva dubia Zetterstedt, 1838, auctt. nec., syn. n.
Melanostoma tschernovi Barkalov, 2009, syn. n.
Melanostoma clausseni Barkalov, 2009, syn. n.

##### Type locality.

The locality of the lectotype is not indicated in the original label. Peck (1988) gave the following information “Svecia'' (= Sweden).

##### Types.

**Lectotype of *Melanostoma mellinum*:** female, pinned, deposited in LSUK. We designate the specimen with collection number LINN 5304 as lectotype of *Musca mellina* Linnaeus, 1758. The lectotype bears no original label. Images: http://www.linnean-online.org/22691/.

**Lectotype of *Syrphus melliturgus*:** male, pinned, deposited in MNHN. Original label: ’Meigen 1482 40 / *Syrphus melliturgus* male’. Of the specimen only the thorax with legs and both wings remains. The identity of the specimen cannot be ascertained, but we accept the synonymy.

The type of *Syrphus minutum* Macquart, 1829 and syntypes of *Syrphus unicolor* Macquart, 1829 apparently exist at Musée d’Histoire Naturelle, Lille, France, but could not be studied.

*Melanostoma mellinum* var. *melanatus* (type material deposited in MZT) is here synonymized with *Melanostoma mellarium*.

The types of *Melanostoma mellinum* var. *angustatoides* Kanervo, 1934 are lost (see section Type studies).

The lectotype of *Scaeva dubia* Zetterstedt, 1838, original label ’S. dubia f Juckasjärvi'(in MZL) was studied, as well as one syntype (see section Type studies).

A male paratype of *Melanostoma clausseni* Barkalov, 2009 from the type locality (Russia, Altai, Ulaganskii region, Kuraiskii, 2500-2800m) was provided for study by A. V. Barkalov, and the taxon is here synonymized with *Melanostoma mellinum*. The type materials of remaining synonyms have not been studied.

##### Additional material studied.

DNA voucher specimens in MZH (Table 1); 85 males and 100 females in MZH; 25 male and 25 female specimens in AHPC.

##### Male.

*Head*: Colour brownish black. Angle of approximation of eyes 80°–90°. Eye contiguity about as long as frontal triangle. Ocellar triangle slightly longer than wide with dark pile and with thin grey dusting. Occiput very narrow and dorsally usually with dark pile and laterally with pale pile. Frontal triangle with indistinct or thin grey dusting. Lateral parts of frontal triangle with dark pile. Face shining with indistinct or thin grey dusting. Lateral parts of face with pile which colour varying from completely pale to almost completely dark. Gena about as wide as basoflagellomere and with greyish dusting. Antenna mainly dark brown, usually basoflagellomere with yellow spot basally at ventral side. Basoflagellomere about 1.4 times as long as its width. Arista usually yellowish brown and about twice as long as basoflagellomere. Longest pile of arista at most half width of arista at base as in Fig. 4B.

*Thorax*: Scutum shining brownish black except for thin greyish dusting at anterior margin. Scutum with erect pile and with shorter semi-adpressed usually mainly pale pile on anterior margin. Pile rarely mainly dark on scutum. Anterior part of scutum almost always with short erect pile which length about fourth part of length of scutellum. Postpronotum totally covered by thin greyish dusting. Notopleuron usually covered by thin greyish dusting. Scutellum shining brownish black. Scutum and scutellum with pile which colour varying from totally pale brown to almost totally dark. Pleura brownish black and usually with thinly grey dusting. Pleura with pale or brownish erect pile. Calypter brownish with pale brownish pile at edge. Halter yellow with slightly darkened base of stem.

*Wing*: Usually completely microtrichose, rarely with small bare area on base of cell BM. Membrane with indistinct brownish ting. Stigma usually yellowish brown.

*Legs*: Coxa black with grey dusting. Trochanter dark brown. Femur usually mainly black except yellow apical part. Tibia usually mainly yellow with dark brown ring varying size. Metatibia usually with a longer dark ring than other tibiae. Tarsus dark brown except mesotarsus with the two basal segments yellow. Leg with pale and dark pile mixed.

*Abdomen*: Terga dark brown or black with weak greyish dusting. Tergum 2 with pair of yellow oval maculae. Terga 3 and 4 with pair of yellow elongated maculae. Terga 1 and 2 laterally with long pale pile. Terga with varying amount of dark and pale semi-adpressed pile outside of yellow maculae. Only pale pile on yellow maculae. Terga 2, 3 and 4 each about as long as wide. Sterna with weak dusting and with pale semi-adpressed pile. Sternum 2 about 1.3 times as long as its width at its posterior margin. Sternum 3 about 1.2 times as long as its width at its anterior margin. Sternum 4 about as long as its width at its anterior margin. Shape of sterna 2–4 are shown in Fig. 5D.

*Male genitalia*: Cercus and surstylus (Fig. 6D). Postgonite short and without distinct ridges laterally (Figs 7D, 8D). Postgonite ventrally in Fig. 9D. The hypandrial margin at postgonites with long triangular projections, index DL less than 1.2 (Figs 10D, 11D).

##### Female.

Similar to male, but differs as follows:

*Head*: Frons shining except greyish dusted triangles. Ventral to the dusted triangles the thinly dusted area is not connected to the sides of lunule. Frons at level of front ocellus slightly narrower than length of antenna. Dorsal part of frons with dark pile and ventral part of frons with pale pile. Occiput as broad as two diameters of an ocellus and usually dorsally with pale and dark pile and laterally with pale pile.

*Thorax*: Scutum and scutellum with short pale pile. Calypter whitish yellow with whitish pile at edge.

*Legs*: Coloration of femur varies from mainly yellow to mainly dark. Metaleg usually largely darker than other leg.

*Abdomen*: Some specimens have all terga dorsally only with pale yellowish semi-adpressed pile. Tergum 2 without or with pair of small yellow oval maculae. Terga 3 and 4 with pair of yellow elongated triangular maculae of varying size or yellow maculae lacking. Tergum 5 at anterior margin without or with pair of short yellow maculae. Tergum 2 about 0.5 times as long as its width at its posterior margin. Tergum 3 about 0.5 times as long as its width at its posterior margin. Tergum 4 about 0.6 times as long as its width at its posterior margin. Sternum 2 about 0.6 times as long as its width at its posterior margin. Sternum 3 about 0.6 times as long as its width at its anterior margin. Sternum 4 about 0.6 times as long as its width at its anterior margin. Sterna 3 and 4 with posterior margin of sternum distinctly broader than width of anterior margin of sternum. Sternum 4 is at most slightly longer than sternum 3. Shape of sterna 2–4 are shown in Fig. 12E, F.

##### Length

(25 males and 25 females): Body 6–8 mm.

##### Distribution.

A very common and abundant species, known from the whole Palaearctic area and North Africa.

**Figure 15.**
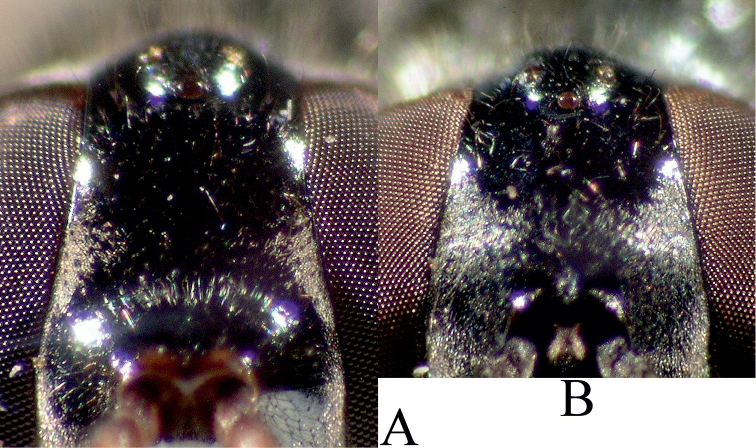
Frons of female. **A** *Melanostoma mellinum* and **B** *Melanostoma certum*.

**Figure 16.**
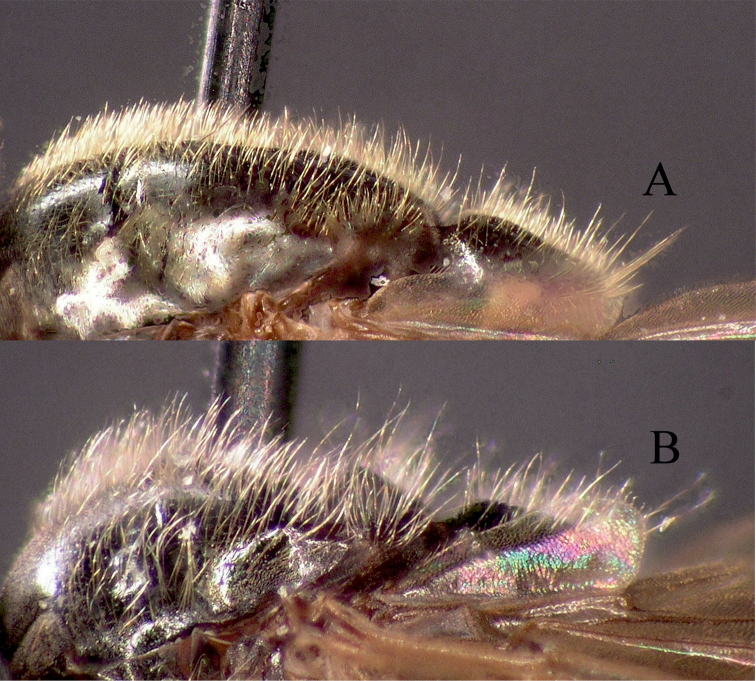
Lateral view of male scutum. **A** *Melanostoma mellinum* and **B** *Melanostoma certum*.

#### Melanostoma scalare

Taxon classificationAnimaliaDipteraSyrphidae

(Fabricius, 1794)

Figs 4A , 5A , 6A , 7A , 8A , 9A , 10A , 11A , 12A , 14A

Syrphus scalaris Fabricius, 1794
Syrphus gracile Meigen, 1822
Syrphus maculosum Meigen, 1822

##### Type-locality.

Fabricius (1794) described this species from “Kiliae'' (= Kiel, Germany).

##### Types.

Types were not studied.

##### Additional material studied.

DNA voucher specimens in MZH from Hungary, Italy, the Netherlands and Finland (MZH); 16 male and 20 female specimens from Luxembourg, Netherlands, Serbia, Sweden (MZH); 25 male and 25 female specimens in AHPC.

##### Male.

*Head*: Colour bluish black. Angle of approximation of eyes 80°–90°. Eye contiguity about as long as length of frontal triangle. Ocellar triangle slightly longer than wide with dark and pale pile and with thin grey dusting. Occiput very narrow and with pale pile. Frontal triangle with dense grey or yellowish grey dusting except area above lunule with thin dusting. Lateral parts of frontal triangle with pale pile. Face with dense grey or yellowish grey dusting except shiny facial tubercle. Lateral parts of face with pale pile. Gena about as wide as basoflagellomere and with dense greyish dusting. Antenna mainly yellow, anterodorsal margin of basoflagellomere distinctly brown. Basoflagellomere about 1.5 times as long as wide. Arista yellowish brown and about twice as long as length of basoflagellomere. Arista short pubescent with pile about as long as width of base of arista (Fig. 4A).

*Thorax*: Scutum shining bluish black except for thin greyish dusting at anterior margin. Scutum with pale yellow erect pile and with shorter pale semi-adpressed pile on anterior margin. Anterior part of scutum with long erect pale pile which length at most third of length of scutellum. Postpronotum totally covered with dense greyish dusting. Notopleuron covered with greyish dusting. Scutellum shining bluish black with pale erect pile at its dorsum, hind margin and ventral side. Pleura bluish black with grey or yellowish grey dusting. Pleura with pale erect pile. Calypter whitish yellow with whitish pile at edge. Halter yellow with slightly darkened base of stem.

*Wing*: Microtrichose except for cell BM basally narrowly bare, with indistinct brownish ting. Stigma pale yellowish brown.

*Legs*: Coxa black with grey dusting. Trochanter yellowish brown. Femur and tibia yellow and brown in varying extent. Tarsus yellowish brown. Metaleg usually darker than other leg. Leg with all pile pale.

*Abdomen*: Terga dark brown or black with weak greyish dusting. Tergum 2 with yellow long oval maculae. Terga 3 and 4 with a pair of long yellow subrectangular maculae. Terga 1 and 2 laterally with long pale pile. Terga with dark and pale semi-adpressed pile. Pale pile on yellow maculae. Terga 2, 3 and 4 each about twice as long as wide. Sterna with weak dusting and with pale semi-adpressed pile. Sternum 2 about 2.5 times as long as its width at its posterior margin. Sternum 3 about twice as long as its width at its anterior margin. Sternum 4 nearly twice as long as its width at its anterior margin. Shape of sterna 2–4 are shown in Fig. 5A.

*Male genitalia*: Cercus and surstylus (Fig. 6A). Postgonite long and without distinct ridges laterally (Figs 7A, 8A). Postgonite ventrally in Fig. 9A. The hypandrial margin at postgonites with short triangular projections, index DL about 1.5 (Figs 10A, 11A).

##### Female.

Similar to male, but differs as follows:

*Head*: Frons shining except greyish dusted triangles which narrowly connected to dusted area of face. Frons at level of front ocellus about as broad as length of antenna. Occiput as broad as two diameters of an ocellus.

*Thorax*: Scutum with short pale pile.

*Abdomen*: Tergum 2 with pair of yellow oval maculae. Terga 3 and 4 with a pair of yellow elongated triangular maculae. Tergum 5 at anterior margin with a pair of short yellow maculae. Tergum 2 about 0.6 times as long as its width at its posterior margin. Tergum 3 about 0.7 times as long as its width at its posterior margin. Tergum 4 about 0.9 times as long as its width at its posterior margin. Sternum 2 about 0.8 times as long as its width at its posterior margin. Sternum 3 about 0.8 times as long as its width at its anterior margin. Sternum 4 about 0.9 times as long as its width at its anterior margin. Shape of sterna 2-4 are shown in Fig. 12A.

##### Length

(25 males and 25 females): Body 7–9 mm.

##### Distribution.

A very common and abundant species, known from the whole Palaearctic area and Northern Africa.

### Differential diagnoses

The species in the genus *Melanostoma* are highly variable in colour and dusting (pollinosity, microtrichosity), and none of the species can be identified solely based on the pale colour and dusting patterns of the abdomen or colouring of legs. The only Fennoscandian species of *Melanostoma* that seems to be quite stable in its coloration is *Melanostoma scalare*, but this taxon is also easily distinguished from its congeners based on other characteristics.

*Melanostoma scalare* can be easily told apart from the other *Melanostoma* species by its pilose arista (Fig. 4A), densely dusted face and long abdomen (Fig. 14B). *Melanostoma certum* sp. n. is the relatively smallest and darkest species of the genus. Males of *Melanostoma certum* can be separated from *Melanostoma mellarium* and *Melanostoma mellinum* by the presence of only long (whitish) pile on scutum (Fig. 16B) while *Melanostoma mellarium* and *Melanostoma mellinum* have short dark or pale yellowish pile on scutum (Fig. 16A). Females of *Melanostoma certum* can be partly separated from *Melanostoma mellarium* and *Melanostoma mellinum* by the combination of the totally dark abdomen and presence of only whitish pile on abdomen. The female specimens of *Melanostoma mellarium* and *Melanostoma mellinum* with pale pilose and dark integument of abdomen (melanic females) have sterna 3 and 4 distinctly broadened towards their posterior margins (Fig. 12D–F), while *Melanostoma certum* has sterna 3 and 4 evenly broad (Fig. 12B–C). Although typical specimens of *Melanostoma mellarium* have shiny, indistinctly greyish dusted pleura, some *Melanostoma mellarium* have thinly, but distinctly, greyish dusted pleura as in *Melanostoma certum* and typical *Melanostoma mellinum*. Lastly, *Melanostoma mellarium* has a longer abdomen than *Melanostoma certum* and *Melanostoma mellinum*. Therefore, a reliable identification of *M. mellarium, M. mellinum* and usually *Melanostoma certum* implies the study of the length and width proportions of terga and shapes of sterna.

### Identification key to North European species of *Melanostoma*

*Males* (external morphological features)

**Table:** 

1	Arista with pile about as long as width of base of arista (Fig. 4A). Abdomen with sternum 2 about 2.5 times as long as its width at its posterior margin and sternum 3 nearly twice as long as its width at its anterior margin (Fig. 5A)	*Melanostoma scalare* (Fabricius, 1794)
–	Arista with pile shorter than half width of base of arista (Fig. 4B). Abdomen with sternum 2 at most twice as long as its width at its posterior margin and sternum 3 at most 1.6 times as long as its width at its anterior margin (Fig. 5B, C, D)	2
2	Anterior part of scutum with long whitish pile at least a third of the length of scutellum (Fig. 16B). Angle of approximation of eyes 85°–100°. Usually terga 2–4 distinctly longer than wide. Sternum 2 at most 1.5 times as long as its width at its posterior margin (Fig. 5B). Sternum 3 at most 1.4 times as long as its width at its anterior margin (Fig. 5B). Pleura usually densely yellowish grey dusted and almost matt	*Melanostoma certum* sp. n.
–	Anterior part of scutum with pile of variable colour and shorter, about a quarter of the length of scutellum (Fig. 16A). Angle of approximation of eyes 80°–90°. If anterior part of scutum with long whitish pile then terga 2–4 about as long as wide	3
3	Usually terga 2–4 about as long as wide. Pleura usually densely yellowish grey dusted and almost matt. Sternum 2 about 1.3 times as long as its width at its posterior margin (Fig. 5D). Sternum 3 about 1.2 times as long as its width at its anterior margin (Fig. 5D). Anterior part of scutum with short mainly pale yellowish pile mixed with variable amount of dark pile	*Melanostoma mellinum* (Linnaeus, 1758)
–	Usually of terga 2–4 distinctly longer than wide. Pleura usually distinctly shining on posterior part of anepisternum, anterior part of anepimeron and dorsal part of katepisternum. Sternum 2 at least 1.6 times as long as its width at its posterior margin (Fig. 5C). Sternum 3 about 1.5 times as long as its width at its anterior margin (Fig. 5C). Anterior part of scutum with variable ratios of short pale yellowish and dark pile	*Melanostoma mellarium* (Meigen, 1822), stat. n.

*Males* (genitalia characteristics)

**Table:** 

1	Index DL more than 2.2 (Figs 10B, 11B)	*Melanostoma certum* sp. n.
–	Index DL less than 1.7 (Figs 10A, C, D, 11A, C, D)	2
2	Postgonite short (Figs 7D, 8D)	*Melanostoma mellinum* (Linnaeus, 1758)
–	Postgonite long (Figs 7A, C, 8A, C)	3
3	Index DL about 1.5 (Figs 10A, 11A). Postgonite without distinct ridges laterally (Figs 7A, 8A)	*Melanostoma scalare* (Fabricius, 1794)
–	Index DL less than 1.2 (Figs 10C, 11C). Postgonite with distinct ridges laterally (Figs 7C, 8C)	*Melanostoma mellarium* (Meigen, 1822), stat. n.

*Females* (external morphological features)

**Table:** 

1	Arista with pile about as long as width of base of arista (Fig. 4A). Cell BM basally without microtrichia. Face except facial knob with distinct grey dusting	*Melanostoma scalare* (Fabricius, 1794)
–	Arista with pile shorter than half width of base of arista (Fig. 4B). Wing almost always entirely covered with microtrichia. Face shining with weak greyish dusting	2
2	Posterior ocellus in front of the hind eye line (Fig. 13A). Abdomen long with nearly parallel sides. Total length of terga 2, 3 and 4 at least 1.9 times as long as width of posterior margin of tergum 3 (Fig. 14C) (Difficult feature because lateral margins of terga turn under abdomen). Tergum 3 usually almost as long as wide. Pleura usually partly distinctly shining on posterior part of anepisternum, anterior part of anepimeron and dorsal part of katepisternum	*Melanostoma mellarium* (Meigen, 1822), stat. n.
–	Posterior ocellus about at the level of the hind eye line (Fig. 13B). Abdomen short, narrowly or broadly oval. Total length of terga 2, 3 and 4 at most 1.7 as long as width of posterior margin of tergum 3 (Fig. 14B, D). Tergum 3 usually distinctly shorter than its width. Pleura usually densely yellowish grey dusted and almost matt	3
3	Terga black without yellow maculae and with whitish semi-adpressed pile. Sterna 3 and 4 almost evenly broad, rarely slightly broadened towards posterior margins (Figs 12B, 12C). Frons thinly dusted laterad of lunule (Fig. 15B)	*Melanostoma certum* sp. n.
–	Terga black with or without yellow maculae and with pale yellowish semi-adpressed pile usually mixed with dark pile. Sterna 3 and 4 with posterior margin of sternum distinctly broader than width of anterior margin of sternum (Figs 12E, 12F). Frons shiny laterad of lunule (Fig. 15A)	*Melanostoma mellinum* (Linnaeus, 1758)

## Discussion

The COI gene 3'–region did not present haplotypes unique to each morphologically identified species, e.g. the *Melanostoma scalare* taxon, which is morphologically well defined, only showed COI haplotypes shared with other taxa (Fig. 2, Table 2). The ITS2 marker, however, was resolved into five unique sequence clusters (Fig. 3). The new morphological characteristics identified for the *Melanostoma* taxa occurring in northern Europe are fully consistent with the information from the ITS2 gene region. Thus, based on the ITS2 spacer region and the congruent morphology, and the type studies discussed above, we recognize four taxa in Northern Europe as follows, *Melanostoma certum* sp. n., *Melanostoma mellinum* (Linnaeus, 1758), *Melanostoma mellarium* (Meigen, 1822), stat. n., and *Melanostoma scalare* (Fabricius, 1794). All species and specimens originating from Russia, Siberia (males and females of *Melanostoma clausseni* Barkalov, *Melanostoma tschernovi* Barkalov, *Melanostoma dubium* and *Melanostoma mellinum*) that were send for molecular study by Dr. A.V. Barkalov (see Table 1) were identified by Dr. A.V. Barkalov and compared with the types and other materials in his possession. The materials included one paratype, a male of *Melanostoma clausseni* Barkalov, other specimens of *Melanostoma clausseni* and *Melanostoma tschernovi* used for molecular study were not types but most specimens originated from areas close to the type localities. Comparison of the external morphology and male genitalia for these materials (including the paratype of *Melanostoma clausseni*) with the specimens of the *Melanostoma* spp. taxa treated in this study, all fit within the morphological variation of *Melanostoma mellinum* and present identical ITS2 marker sequences. The descriptions of the *Melanostoma clausseni* and *Melanostoma tschernovi* species do not describe differences of male genitalia between these taxa, nor do the descriptions indicate genitalia differences with *Melanostoma dubium* or *Melanostoma mellinum*. Barkalov (2009) includes a key to *Melanostoma dubium*, *Melanostoma clausseni* and *Melanostoma tschernovi* based on external morphological characters only, and these taxa were not compared with *Melanostoma mellinum*. We have established the new synonymies based on our findings.

The process of delimiting and identifying species is potentially better understood if based on comprehensively studied morphology in conjunction with information from DNA sequences of independent loci, and including samples/specimens from as broad geographical distributions as possible. This approach was possible in this particular group as most of the studied taxa of this group are abundant and widely distributed, but only morphology and one genetic marker agree while the COI gene fragment was proved to be uninformative. A high number of haplotypes for the 3'–fragment of the COI gene was recorded (Table 2). Most species exhibited shared haplotypes with another species (Table 2). This could result from incomplete lineage sorting in a recently diverged taxon and / or mitochondrial introgression. The hypothesis of incomplete lineage sorting is plausible since ancestral variability may have been maintained in Europe where the taxa of the genus are widely distributed and copious.

The nuclear ITS2 gene region is still less applied than mtDNA genes (e.g. COI, COII, cytB) for resolving or delimiting closely related taxa. We found that the ITS2 amplified well only for ‘fresh'specimens of <3 years. In this study the ITS2 marker provided complete concordance with our independently established morphological hypothesis for North European *Melanostoma* spp. Sonet et al. (2012) in their multilocus study on the calliphorid species pair *Lucilia caesar* (Linnaeus, 1758) and *Lucilia illustris* (Meigen, 1826) found a high number of intraspecific haplotypes for each of the studied mtDNA genes (COI, COII, 16S) and the highest number of haplotypes for the COI gene. They also sequenced the nuclear ribosomal 28S and the ITS2 regions, and found only 1–2 genotypes per species for these. Our results parallel theirs with a high number of haplotypes for the mtDNA gene regions, and a low number for the nuclear ribosomal cluster markers. We cannot, however, exclude the possibility of finding shared genotypes of the ITS2 marker also for *Melanostoma* spp. when including additional material. For the identification of recently diverged species of Syrphinae the ITS2 marker appears informative and in the present study, found superior to the COI gene as to its information content.

That integumental expression of pale (yellow to red) colour patterns of hoverfly abdomen can be temperature dependent as shown for taxa of *Eupeodes* Osten Sacken, 1877 (Dušek and Láska 1974). They demonstrated that adult colours became darker with decreasing temperatures experienced during pupal development. Heal (1989) showed that pigmentation of adult *Eristalis tenax* (Linnaeus, 1758) specimens was influenced by the temperature experienced during pre-imaginal stages in captive rearing, where specimens showed less light pigmentation at lower temperatures. Recently, Wright and Skevington (2013) obtained the same result at laboratory rearing of Australian species of *Episyrphus*, especially for *Episyrphus viridaureus* (Wiedemann, 1824). Since the body temperature of adult syrphids has a direct effect on their activity (Gilbert 1985), such colour pattern plasticity has been explained in an adaptive context and associated with thermoregulation (Heal 1981; Holloway 1993). This fits well with the observation that the frequency of *Melanostoma* spp. with completely dark abdomen (melanic female specimens of *Melanostoma mellinum* and female of *Melanostoma certum*) is higher at higher latitudes, as a dark coloration of insect integument and pilosity maximizes the potential to absorb solar radiation for body heating. Dark females may be able to remain active for longer than bright coloured individuals (MacGowan et al. 1997). Nedeljković et al. (2013) found that the syrphid *Chrysotoxum tomentosum* Giglio-Tos, 1890, which occurs at higher altitudes, is darker in coloration than its sibling species *Chrysotoxum festivum* (Linnaeus, 1758), which appears at lower altitudes. A similar pattern is described for other syrphine genera with sibling species pairs, such as *Melangyna* Verrall, 1901 (*Melangyna quadrimaculata* (Verrall, 1873) and *Melangyna umbellatarum* (Fabricius, 1794)) (Rotheray and Gilbert 2011).

## Supplementary Material

XML Treatment for Melanostoma

XML Treatment for Melanostoma certum

XML Treatment for Melanostoma mellarium

XML Treatment for Melanostoma mellinum

XML Treatment for Melanostoma scalare
